# Capturing the extensive diversity of marine anaerobic scuticociliates (Oligohymenophorea, Ciliophora) through cultivation

**DOI:** 10.1007/s42995-025-00350-5

**Published:** 2026-03-30

**Authors:** Kateřina Koštířová, Johana Rotterová, William A. Bourland, Ivan Čepička

**Affiliations:** 1 Department of Zoology, Faculty of Science, Charles University, Viničná 7, Prague, 12800 Czech Republic; 2 Department of Marine Sciences, University of Puerto Rico Mayagüez, Mayagüez, PR USA

**Keywords:** 18S rRNA gene, Anaerobiosis, Protargol impregnation, Scanning electron microscopy, Scuticociliatia

## Abstract

Marine anoxic sediments are expansive ecosystems, effectively devoid of oxygen, where eukaryotic life is predominantly represented by protists. In this study, we surveyed a range of such habitats and uncovered novel diversity within ciliated protists from the subclass Scuticociliatia (class Oligohymenophorea). We establish three new genera of marine anaerobic scuticociliates within the family Anaerocyclidiidae that were previously detected exclusively through cultivation-independent environmental surveys. Our results show that marine Anaerocyclidiidae have a global distribution and occur frequently in anoxic sediments. Notably, all studied marine Anaerocyclidiidae host prokaryotic ectosymbionts of varying sizes and shapes, potentially representing distinct prokaryotic lineages. Through broad geographic sampling and the establishment of the largest culture collection of marine anaerobic scuticociliates to date, we investigated the diversity, morphology, behavior, and symbiotic associations within this significant ciliate lineage. These findings highlight the importance of cultivation approaches to uncover novel protistan taxa and their symbiotic relationships, expanding our understanding of biodiversity and ecosystem functioning in oxygen-depleted habitats.

**Supplementary Information:**

The online version contains supplementary material available at 10.1007/s42995-025-00350-5.

## Introduction

Hypoxic and anoxic sediments are known to host complex microbial communities, including ciliates, which are often the major bacterial grazers. Anaerobic ciliates are successful in exploiting various oxygen-depleted niches, including freshwater (e.g., ponds, lakes, wetlands, rice fields), brackish (e.g., river estuaries, coastal marshes), and marine sediments (both shallow and deep-water). Discoveries of new anaerobic ciliate lineages have shown that the anaerobic lifestyle is common and has evolved in most ciliate classes (Campello-Nunes et al. 2020; Orsi et al. 2012a, b; Pomahač et al. 2023; Rotterová et al. 2020). One such example is the subclass Scuticociliatia (class Oligohymenophorea), a diverse group of ciliates found in both freshwater and marine habitats. While scuticociliates are mostly represented by aerobic taxa, a number of anaerobic scuticociliate lineages have also been identified, including the recently erected family Anaerocyclidiidae, a diverse lineage predominantly found in freshwater habitats (Poláková et al. 2023). Environmental sequencing and single-cell omics studies have served to provide important insights into the evolution and diversity of ciliates across a range of environments, including extreme habitats, such as anoxic ecosystems (Chen et al. 2021; Feng et al. 2020; Rossi et al. 2019; Sieracki et al. 2019; Zhang et al. 2024). Importantly, many environmental sequencing surveys of anoxic habitats have recovered scuticociliate sequences, indicating that these ciliates are both common and widely distributed, particularly in marine environments (Bernhard et al. 2014; Coyne et al. 2013; Edgcomb et al. 2011a; Kataoka and Kondo 2019; Pasulka et al. 2016; Stock et al. 2009; Stoeck et al. 2003, 2009; Takishita et al. 2010; Wylezich and Jürgens 2011). Nonetheless, traditional cultivation approaches continue to play an essential role, significantly advancing our understanding of protist biology and providing foundational knowledge that complements environmental and omics data (del Campo et al. 2024). Cultivation enables the linking of molecular data with morphology, physiology, behavior, and ecology, offering a more comprehensive understanding of the biology of microorganisms, and can also provide crucial evidence regarding their anaerobic lifestyle. However, relevant morphological data linked to molecular information are, to date, missing for the marine anaerobic scuticociliates.

Anaerobic scuticociliates are common and often abundant in the environment, suggesting that they are important members of microbial eukaryotic communities in anoxic ecosystems (Edgcomb and Pachiadaki 2014; Hu et al. 2024; Orsi et al. 2012a, b; Wylezich and Jurgens 2011). However, their small size and often similar appearances make them easy to overlook and challenging to distinguish morphologically. Multiple molecular markers have been used previously to study scuticociliate diversity (Gao et al. 2012a, 2013, 2014; Liu et al. 2020, 2025; Zhang et al. 2019). However, the 18S rRNA gene, alone, has proven to be a suitable marker, providing sufficient phylogenetic signal to capture both intra- and interspecific variability (Gao et al. 2010, 2012b; Pan et al. 2020; Poláková et al. 2023).

Symbiosis between anaerobic ciliates and prokaryotes is widespread in oxygen-depleted environments (reviewed in Rotterová et al. 2022). A common form of symbiosis is syntrophy, in which the mitochondrion-related organelles (typically hydrogen-producing mitochondria or hydrogenosomes) of a eukaryote host serve as a source of hydrogen for hydrogen-scavenging prokaryotes, such as methanogenic archaea, sulfate-reducing bacteria, or denitrifying bacteria (Beinart et al. 2018; Edgcomb et al. 2011b; Fenchel and Ramsing 1992; Hirakata et al. 2020; Méndez-Sánchez et al. 2024; Schrecengost et al. 2024; Speth et al. 2024). However, the phylogenetic and functional diversity of the putative symbionts in syntrophic relationships is probably greatly underestimated, with many yet to be discovered, especially in marine habitats. In addition to intracellular symbionts, ectosymbionts are also known to occur on the cells of anaerobic scuticociliates isolated from marine and sulfide-rich habitats (Esteban and Finlay 1994; Edgcomb and Pachiadaki 2014; Epstein et al. 1998; Fenchel and Finlay 1991; Orsi et al. 2012a, b; Radek 2010). Yet, the lack of long-term cultures of these organisms has hampered detailed comparative analyses of host specificity and coevolution of the interactions.

In this study, we conducted an extensive analysis of 44 cultured scuticociliate strains isolated from various marine hypoxic/anoxic habitats, utilizing a combination of in vivo observation, silver impregnation methods, transmission and scanning electron microscopy, and molecular approaches based on 18S rRNA gene sequences to describe the diversity of marine lineages that have, so far, been represented only by environmental sequences. We expand the family Anaerocyclidiidae to encompass five fully supported clades of marine and freshwater anaerobic scuticociliates represented by four genera and nine species, namely *Anaerocyclidium* and three newly described marine genera, *Neocyclidium* n. gen., *Metacyclidium* n. gen., and *Maricyclidium* n. gen. Members of the family Anaerocyclidiidae form an independent lineage of anaerobic scuticociliates phylogenetically distinct from Pleuronematida based on 18S rRNA gene sequences.

## Materials and methods

### Sample collection and cultivation

**Fig. 1:**
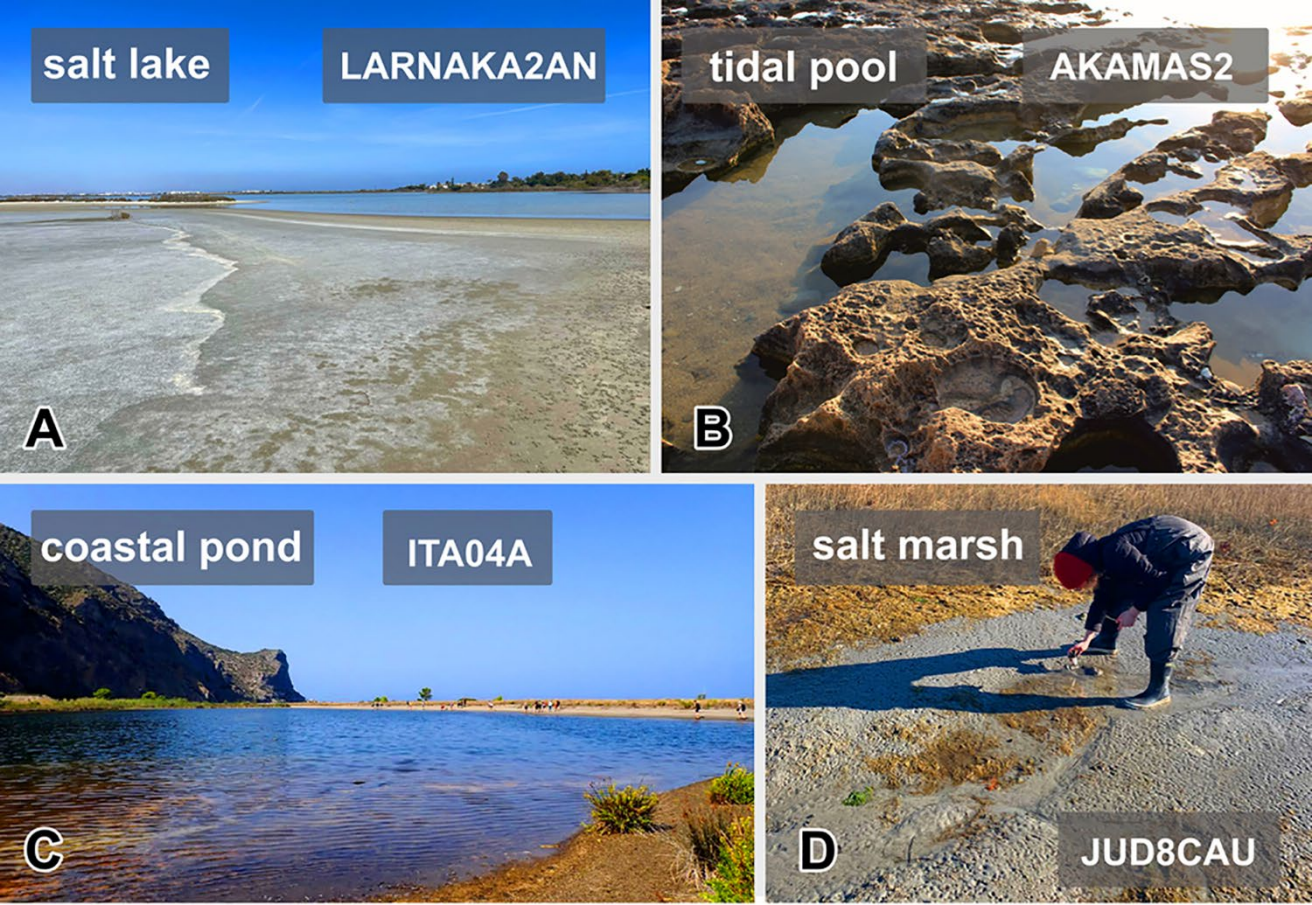
Examples of sampling sites (**A–D**). **A** Salt lake in Larnaka, Cyprus (sampling site of *Maricyclidium* lineage 2 strain LARNAKA2AN). **B** Tidal pool in Cyprus (sampling site of *Metacyclidium pallium* strain AKAMAS2).** C** Coastal pond in Laghetti di Marinello, Italy (sampling site of *Metacyclidium pallium* strain ITA04A).** D** Point Judith Pond salt marshes in Narragansett, Rhode Island, USA (sampling site of *Metacyclidium pallium* strain JUD8CAU). Photo credits: Kristýna Poláková (**A**), Jitka Štumpová (**C**), and Anna Schrecengost (**D**)

Samples were collected in 50 mL Falcon tubes from brackish/marine anoxic or microoxic sediments at various locations worldwide (Fig. 1, Supplementary Table S1). Deep-water samples were collected from sediments during two research cruises: 1) sediment push cores were taken with ROV Hercules on E/V *Nautilus* cruise NA075 at 33°38′24.0"N 118°48′01.3"W at 899 m depth in 2016. After core material was homogenized, sediment subsamples were removed and stored at 4 °C until cultivation, and 2) aboard R/V *Robert Gordon Sproul* (Scripps Institution of Oceanography) in July 2022, from the Santa Barbara Basin, California, USA (34°18′07.0"N 120°00′56.1"W) at a depth of 556 m, using a Soutar boxcorer as described in Gomaa et al. (2024).

Non-clonal cultures were established by inoculating approximately 2 mL of the field samples into a 15 mL Falcon tube with 10 mL of fresh seawater media. Isolated ciliate strains were cultured alongside the original microbial community including unknown prokaryotes and, in some cases, also other anaerobic protists, such as diplomonads, psalteriomonadid heteroloboseans, metopid, or plagiopylean ciliates. Culture tubes were tightly closed to avoid oxygen exchange. Marine strains were cultured in ATCC medium 1525 (Sonneborn 1970). Brackish strains were cultured in a 1:1 mixture of Sonneborn´s *Paramecium* medium (ATCC medium 802) (Sonneborn 1970) and ATCC medium 1525. Cultures were stored in the dark at room temperature and inoculated every second or third week by transferring 1–2 mL of sediment to fresh media. Selected strains were cultured in parallel under anaerobic conditions in a 2.5 L AnaeroJar container with AnaeroGen sachets (Oxoid, UK).

### Morphologic and ultrastructure methods

Living cells were observed at magnifications of 40–1000 × with differential interference contrast illumination using an Olympus BX 53 microscope. Photomicrographs were made with a Canon 6D camera. Video imaging was done using a Canon 80D camera. Protargol (Polysciences Inc., USA, no longer commercially available), wet silver nitrate impregnation, and scanning electron microscopy were done according to Foissner (2014). Samples for transmission electron microscopy were processed as follows: samples were fixed in 2.5% glutaraldehyde and postfixed in 1% osmium tetroxide; after washing, dehydration, and resin embedding, ultrathin sections were contrasted with uranyl acetate and embedded in EPON-Araldite. Prepared specimens were observed in TEM JEOL 1011 transmission electron microscope. Specimens for scanning electron microscopy were observed in a JEOL 6380 LV scanning electron microscope. In vivo measurements were made from microphotographs with calibrated imaging software (ImageJ). Terminology is mainly according to Lynn (2008).

### Single cell methods, DNA extraction, amplification, and sequencing

Genomic DNA was extracted either from hand-picked and washed individual cells, using the MasterPure™ CompleteDNA and RNA Purification Kit (Lucigen, USA), or from about 100 cells in case of monoeukaryotic cultures, using the Genomic DNA Minikit (Geneaid, Taiwan) according to the manufacturer´s instructions. The One*Taq* DNA polymerase (New England Biolabs, USA) was used to amplify the 18S rRNA gene with the following primer combinations: EukA (5'-AACCTGGTTGATCCTGCCAGT-3') and EukB (5'-TGATCCTTCTGCAGGTTCACCTAC-3') with an annealing temperature of 55 °C (Medlin et al. 1988); EukA and ScutiR1 (5'-TCTCTCAATCTGTCAATCCCACT-3') (Poláková et al. 2021) with an annealing temperature of 60 °C; F82 (5'-GAAACTGCGAATGGCTC-3') and EK1498R (5'-CACCTACGGAAACCTTGTTA-3') (Jerome et al. 1996; Marande et al. 2009) with an annealing temperature of 55 °C. Purification of PCR products was done using the Gel/PCR DNA fragments extraction kit (Geneaid, Taiwan) or ExoSAP-IT™ PCR Product Cleanup (Thermo Fisher, USA) according to the manufacturers' instructions. Sanger sequencing was performed on the ABI PRISM 3100 sequencer (Applied Biosystems, USA) at the Laboratory for DNA sequencing, Faculty of Science, Charles University.

### Phylogenetic analyses

The 18S rRNA gene data set consisted of 44 newly determined sequences and 144 sequences from representatives of the seven oligohymenophorean subclasses and the CONThreeP outgroup (one colpodean and two nassophorean sequences) downloaded from GenBank (https://www.ncbi.nlm.nih.gov/nucleotide/). Sequences were aligned using the MAFFT 7 server (Katoh et al. 2002) with the G-INS-i algorithm (http://mafft.cbrc.jp/alignment/server/). The alignment was edited using AliView (Larsson 2014) to trim primer sequences. No other positions were removed. The final alignment consisted of 1779 positions. Phylogenetic trees were constructed by maximum-likelihood (ML) and Bayesian methods. ML analysis was performed in RaxML-NG (Kozlov et al. 2019) under the GTR + Γ model. The best-fit nucleotide substitution model for BI was chosen according to the Akaike Information criterion in ModelGenerator v0.85 (Keane et al. 2006). Branch support was assessed by ML with 1000 bootstrap pseudoreplicates. Convergence was assessed by weighted Robinson–Foulds distance (WRF). Bootstopping test converged after 650 pseudoreplicates. Bayesian analysis was performed using MrBayes 3.2.2 (Ronquist et al. 2012) using the GTR + Γ + I model. Four MCMCs were run for 15,000,000 generations, with a sampling frequency of 1000 generations. The first 25% of trees were removed as burn-in. The average standard deviation of split frequencies after 15 million generations was 0.006. Convergence was also assessed using RWTY (Warren et al. 2017). Trees were viewed in FigTree v1.4.4 (

https://github.com/rambaut/figtree/re- leases/tag/v1.4.4). Uncorrected p-distances were calculated using MEGA 11.0.13 (Tamura et al. 2021). AU test was done using IQ-TREE3 (Wong et al. 2025).

## Results

### Cultivation of anaerobic marine scuticociliates

We successfully established cultures of scuticociliates from various anoxic marine habitats (beach sediments, coastal marshes, and deep-water sediments) primarily focusing on locations in the Mediterranean Sea and along the U.S. Atlantic coast (Fig. 1). However, the global distribution of anaerobic scuticociliates is further indicated by several samples collected from the Indian and Pacific Oceans (Supplementary Fig. S1). We established stable cultures of 44 strains representing most known environmental lineages of marine anaerobic scuticociliates, namely, environmental scuticociliate lineages I, II, and IV sensu Poláková et al. (2023). Notably, target scuticociliates were consistently abundant in freshly collected samples indicating their abundance in natural environments.

### Morphological descriptions and Taxonomic summary

**ZooBank registration of the work:** urn:lsid:zoobank.org:pub:0014237C-DEB8-4E12-9F40-FEDC52DE8436.

**Taxonomic assignment:** Eukaryota: SAR: phylum Ciliophora: subphylum Intramacronucleata: class Oligohymenophorea: subclass Scuticociliatia: family Anaerocyclidiidae Poláková et al. 2023

**Emended diagnosis:** Small, anaerobic, *Cyclidium*-shaped Scuticociliata, phylogenetically distinct from Pleuronematida, with continuous or interrupted somatic kineties and bearing either ecto- or endosymbiotic prokaryotes.

***Neocyclidium*** **n. gen.**

**ZooBank registration:** urn:lsid:zoobank.org:act:5ABFAFFB-BADB-4F45-B803-9B36A8DC12AD.

**Diagnosis:** With characters of the family. Anterior part of uninterrupted bipolar somatic kineties composed of dikinetids and posterior part of monokinetids. Prokaryotic ectosymbionts present.

**Type species: ***Neocyclidium profundum* n. sp.

**Etymology:** Compound of the Greek adjective *néos* (new) and the genus name *Cyclidium*, referring to a newly recognized *Cyclidium*-like genus. Neuter gender.

***Neocyclidium profundum*** **n. sp.**

**ZooBank registration:** urn:lsid:zoobank.org:act:D21C3126-A7EA-42A5-8F6F-8C1B2B277D6C.

**Diagnosis:** Body size about 28 × 14 µm in vivo. Ovoidal, more narrowed anteriorly. Eight uninterrupted bipolar somatic kineties, posterior two-thirds of each consists of monokinetids, anterior one-third of closely spaced dikinetids. Anterior part of somatic kinety 1 very close to, and parallel with paroral.

**Etymology:** Latin adjective *profundum* (deep), referring to the deep-water origin of the type population.

**Type locality:** Deep-water marine sediment sampled during a research cruise aboard R/V *Robert Gordon Sproul* (Scripps Institution of Oceanography) in July 2022, from the Santa Barbara Basin, California, USA (34°18′07.0"N 120°00′56.1"W) at a depth of 556 m.

**Fig. 2:**
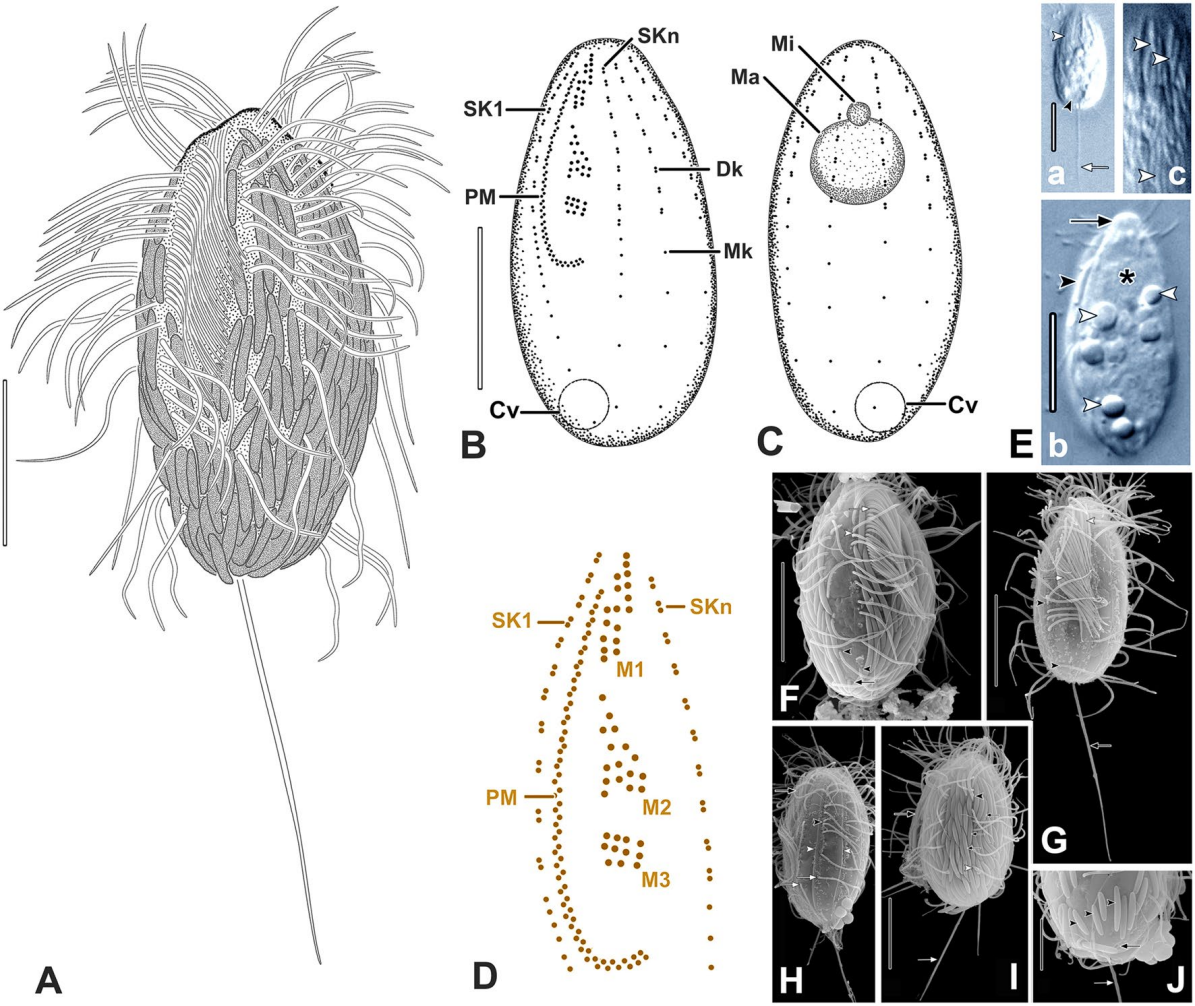
*Neocyclidium profundum* n. gen., n. sp. from life (**A, E**), protargol-impregnated (**B–D**), and in SEM (**F–J**). **A** Typical individual. **B, C** Infraciliature of holotype. **D** Buccal region schematic. **Ea** View of cilia of PM (white arrowhead), CV (black arrowhead), and caudal cilium (white arrow). **Eb, c** View of apical plate (black arrow), symbiont (black arrowhead), cytoplasmic globules (white arrowheads), and Ma (asterisk). **Ec** Ectosymbionts (white arrowheads). **F** View of cilia of PM (white arrow), Dk of SK1 (white arrowhead), posterior Mks (black arrowheads), and excretory pore at SK2 (black arrow). **G** View of cilia of M1 (white arrowhead), PM (white arrow), posterior SK1 (black arrowheads), and caudal cilium (black arrow). **H** View of cortical strips (white arrowheads), Dk of SKn-1 (black arrowhead), posterior Mks (white arrows), and PM cilia (black arrow). **I** View of symbionts (small black arrows), somatic cilia, and caudal cilium (white arrow). **J** Posterior end with symbionts (black arrowheads), excretory pore (black arrow), and caudal cilium (white arrow). *Cv* contractile vacuole, *Dk* somatic dikinetid, *M1–M3* adoral membranelles, *Ma* macronucleus, *Mi* micronucleus, *Mk* somatic monokinetid, *PM* paroral, *SK1* somatic kinety 1, *SKn* somatic kinety n. Scale bars: 10 µm (**A–C**, **E–I**), 5 µm (**D**, **J**)

**Type material:** One slide with the protargol-impregnated holotype and several paratypes of strain M2DEEP is deposited in the collection of the National Museum of the Czech Republic, Prague, inventory number P6E 5596. The holotype is marked with a black ink circle and shown in Fig. 2.

**Gene sequence:** Partial 18S rRNA gene sequences from *Neocyclidium profundum* n. sp., strains M2DEEP, SMALLPO6, SALT61M, and NAO75, were deposited in GenBank with accession numbers PQ800174, PQ800175, PQ800176, and PQ800177, respectively.

**Description (**based on population M2DEEP; Fig. 2; Table 1; Supplementary Fig. S1)**:** Body size in vivo 24–31 × 13–15 µm, length–width ratio about 2:1, 17–26 × 8–13 µm, length–width ratio about 2:1 in protargol preparations. Cell shape ovate, more narrowed anteriorly, apical plate knob-like (Fig. 2Eb). Cells colorless. Single globular macronucleus about 4.8 µm in diameter, usually located in anterior one-third of cell (Fig. 2C; Supplementary Fig. S1). Single micronucleus about 1.7 µm in diameter, adjacent to macronucleus (Fig. 2C). Extrusomes not seen. Contractile vacuole terminal, excretory pore at posterior end of somatic kinety 2 (SK2) (Fig. 2Ea, J). Cytoproct not identified. Cytoplasm with numerous 1–5–2.5 µm globules (Fig. 2Eb). Cells covered with longitudinally attached ectosymbiotic prokaryotes, about 5.3 µm long rods in vivo (Fig. 2A, Ec, I, J). Cells swim moderately fast.

**Table 1:** Morphometric data for *Neocyclidium profundum* (strain M2DEEP)

Characteristics^a^	Method	Mean	*M*	SD	CV	Min	Max	*n*
Cell length	in vivo	28.4	28.2	1.9	6.5	23.6	31.4	18
	*P*	20.6	20.3	2.2	10.6	17.4	25.7	30
Cell width	in vivo	14.4	14.5	0.7	4.6	12.8	15.4	18
	*P*	10	9.8	1.5	14.6	7.6	13.4	30
Cell length–width, ratio	in vivo	2	2	0.1	6.1	1.8	2.2	18
	*P*	2.1	2.1	0.2	9.2	1.8	2.5	30
Buccal region, length	in vivo	13.3	13.3	0.2	1.1	13.2	13.5	3
	*P*	11.2	11.2	1.2	10.5	9	12.9	8
Buccal region length/cell length %	in vivo	50	50	0.0	9.1	50	60	3
	*P*	50	50	0.1	10.5	50	70	8
Buccal region, width	*P*	2.5	2.5	0.1	5.9	2.4	2.6	2
Somatic kineties, number	*P*	8.0	8.0	0.0	0.0	8.0	8.0	10
Kinetids in mid-dorsal kinety, number	*P*	13	13	1.1	8.1	12	14	10
Distance between M3 and posterior end of the paroral	*P*	4.1	4.1	0.0	0.0	4.1	4.1	1
Transverse distance between mid-dorsal kineties	*P*	2.7	2.6	0.5	17.5	2.2	3.6	8
Somatic cilia, length	in vivo	7.6	7.8	0.5	6.6	6.6	8.2	10
Caudal cilium, length	in vivo	17.4	17.6	0.7	4.2	16.0	18.1	10
Macronucleus, diameter	*P*	4.8	4.7	0.8	16.2	3.6	6.2	28
Micronucleus, diameter	*P*	1.7	1.8	0.3	19.9	1.3	2.1	7
Ectosymbionts, cell length	in vivo	5.3	5.1	1.2	22.0	3.9	8.5	18
	SEM	3.3	3.3	0.5	15.7	2.7	4.3	15

^a^All distances in µm. Measurements made using ocular micrometer

*CV* coefficient of variation (%), *M* median, *M1–M3* adoral membranelle 1–3, *Max* maximum value, *Mean* arithmetic mean, *Min* minimum value, *n*, number of cells studied, *P* protargol, *SD* standard deviation of the arithmetic mean, *SEM* scanning electron microscopy

Somatic cilia about 7.6 µm long, distributed evenly over the cell body in eight bipolar somatic kineties, posterior two-thirds of each somatic kinety composed of monokinetids, anterior one-third of each composed of closely spaced dikinetids (Fig. 2B, C). Single caudal cilium, usually shorter than body length (about 17 µm) to nearly as long as body length as observed in SEM preparations (Fig. 2A, Ea, G, I, J).

Buccal area extends about 50% of body length. Paroral membrane reverse J-shaped, about 13 µm long in vivo (Fig. 2B, D; Supplementary Fig. S1A, E, F). Adoral membranelle one (M1) consists of three longitudinal rows of basal bodies of different length, M2 shorter, rectangular, M3 consists of two or three horizontally arranged rows basal bodies (Fig. 2D; Supplementary Fig. S1). Densely spaced monokinetids in midportion of SK1. Anterior part of SK1 very close to, and parallel with paroral (Fig. 2D, F; Supplementary Fig. S1). Wide postoral bare area between SK1 and SKn (Fig. 2B; Supplementary Fig. S1). Scutica not identified. No discernible cytopharyngeal basket.

**Ecology and occurrence:** Marine/brackish habitat. Found in deep-water sediments, brackish lake sediments, and salt marsh sediments. Isolates from both the Pacific and Atlantic coasts.

**Remarks:**

Other populations of *N. profundum* were not studied in detail with silver impregnation methods.

***Metacyclidium*** **n. gen.**

**ZooBank registration:** urn:lsid:zoobank.org:act:4E70E00B-8428-4E15-BAB3-BFBFF29A77AD.

**Diagnosis:** With characters of the family. Uninterrupted bipolar somatic kineties composed of dikinetids anteriorly and posteriorly with intervening segment composed of monokinetids. Extrusomes present. Prokaryotic ectosymbionts present.

**Type species:** *Metacyclidium pallium* n. sp.

**Etymology:** Compound of the Greek prefix *meta-* (after) and the genus name *Cyclidium*, referring to a newly identified genus resembling *Cyclidium* but distinct from it. Neuter gender.

***Metacyclidium pallium*** **n. sp.**

**ZooBank registration:** urn:lsid:zoobank.org:act:53BE30C3-C762-42A9-9556-F0AE610A8413.

**Diagnosis:** Body size about 24 × 11 µm in vivo. Cell shape in vivo ovoidal, only slightly narrowed anteriorly and broadly rounded posteriorly. Seven or eight uninterrupted bipolar somatic kineties, consist of dikinetids anteriorly and posteriorly with short intervening segment of monokinetids. Closely spaced kinetids of anterior part of somatic kinety one parallel with and very close to paroral. Extrusomes scant, narrowly ellipsoidal, about 2 × 0.5 µm. Ectosymbionts of two morphotypes: large broadly fusiform and short, slender cylindroidal.

**Etymology:** Latin noun *pallium* (mantle, cloak) in apposition, referring to the dense layer of ectosymbionts covering the ciliate cells.

**Type locality:** Tidal pool sediment on a rocky beach, South Akamas beach, Cyprus (35°02′27.8"N 32°16′36.4"E).

**Fig. 3:**
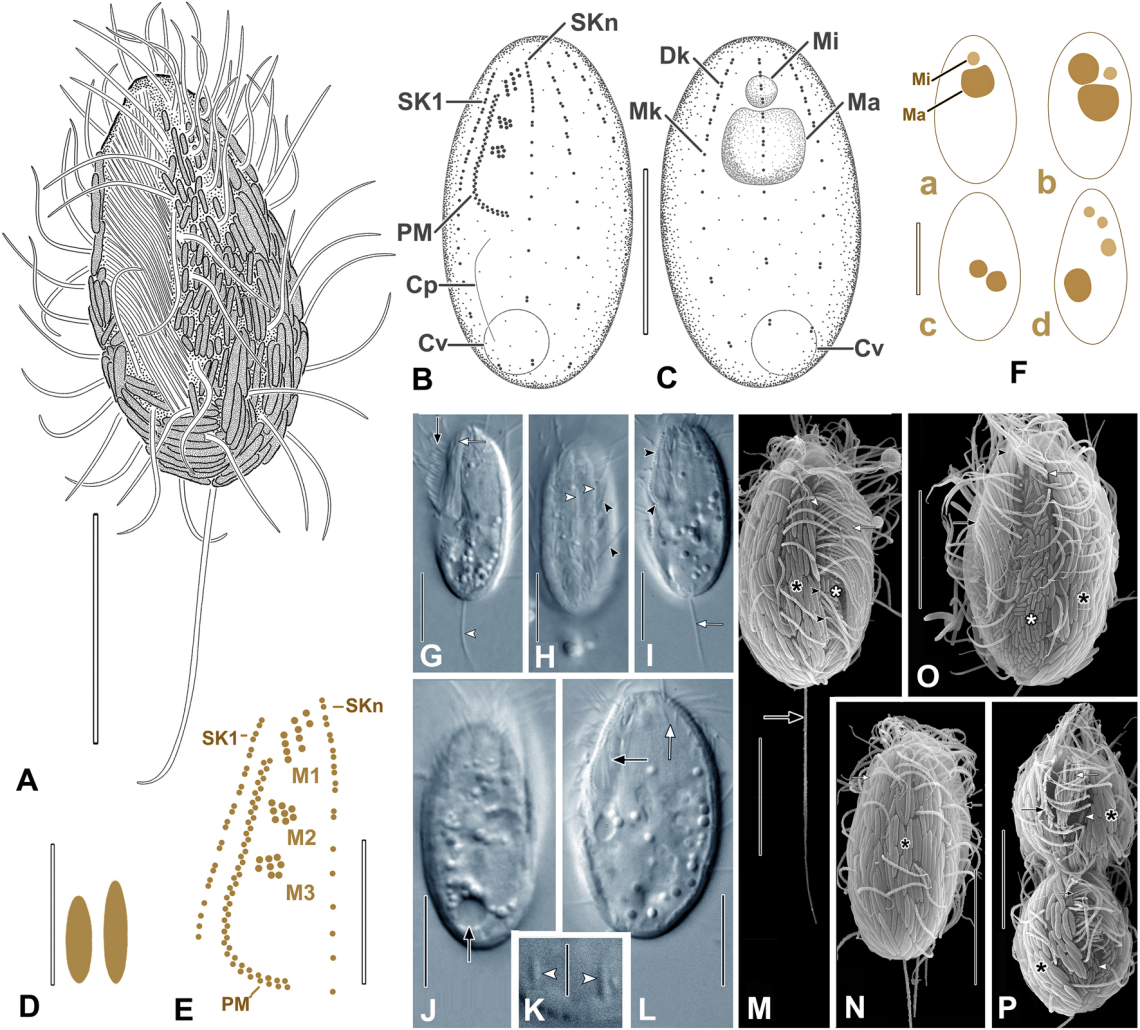
*Metacyclidium pallium* n. gen., n. sp. from life (**A, D, G–L**), protargol (**B, C, E, F**), and in SEM (**M–P**). **A** Typical cell. **B, C** Holotype. **D** Extrusomes. **E** Buccal region. **F** Nuclear apparatus. **G** PM cilia (black arrow), M1 (white arrow), and CC (arrowhead). **H** Somatic cilia (black arrowheads) and SKs (white arrowheads).** I** PM (arrowheads) and CC (arrow). **J** CV in diastole.** K** Extrusomes. **L** Extrusomes (white arrow) and buccal cavity (black arrow). **M** Large (black asterisk) and small (white asterisk) symbionts, Dk of SK1 (white arrowhead), PM (white arrow), and CC (black arrow). **N** PM cilia (black arrow), Dk (white arrow), and symbionts (asterisk). **O** Symbiont types (asterisks), PM (black arrow), Dk of SKn–1 (white arrow), and M1 (black arrowhead). **P** Divider with small (arrowhead) and large (asterisk) symbionts, PM (black arrows) and M1–M3 (white arrow). ***CC*** caudal cilium, *Cp* cytoproct, *Cv* contractile vacuole, *Dk* dikinetid, *M1–M3* adoral membranelles, *Ma* macronucleus, *Mi* micronucleus, *Mk* monokinetid, *PM* paroral, *SK1* somatic kinety 1, *SKn* somatic kinety n. Scale bars: 10 µm (**A–C**, **F–J**, **L–P**); 5 µm (**E**, **K**); 2 µm (**D**)

**Type material:** One slide with the protargol-impregnated holotype and several paratypes of strain AKAMAS2 is deposited in the collection of the National Museum of the Czech Republic, Prague, inventory number P6E 5597. The holotype is marked with a black ink circle and shown in Fig. 3.

**Gene sequence:** Partial 18S rRNA gene sequences from *Metacyclidium pallium* n. sp., strains AKAMAS2, 104BAHNO, JUD8CAU, LUC3, RIDKAN, ITA04A, and SIPPWOOD, were deposited in GenBank with accession numbers PQ800178, PQ800179, PQ800180, PQ800181, PQ800182, PQ800183, and PQ800184, respectively.

**Description** (based on population AKAMAS2; Fig. 3; Table 2; Supplementary Fig. S2, video 1)**:** Body size in vivo 21–27 × 9–12 µm, length–width ratio about 2.3; size in protargol preparations 16–22 × 8–12 µm, length–width ratio about 2. Cells in vivo ovoidal, only slightly rounded anteriorly and broadly rounded posteriorly with conspicuous cortical furrows and apical plate (Fig. 3G–I). One or two globular macronuclei (about 4 µm in diameter), usually in anterior one-half of cell, chromatin homogeneous (i.e., without distinct nucleoli) in protargol preparations (Fig. 3C, Fa–d). One-to-three spherical micronuclei (about 1.5 µm in diameter), usually adjacent to macronuclei (Fig. 3C, Fa–d). Contractile vacuole subterminal in right posterior one-fifth of cell (Fig. 3J). Cytoplasm colorless with scattered refractive globules about 1.5 µm in diameter (Fig. 3G, J, L). Food vacuoles about 3 µm in diameter (Fig. 3G, J, L). Extrusomes scant, fusiform, about 2 × 0.5 µm (Fig. 3D, K, L). Cells covered with longitudinally arranged ectosymbiotic prokaryotes of two different morphotypes: larger, narrowly ellipsoidal (about 3–4 × 0.6 µm) and smaller, cylindroidal (about 1.5 × 0.3 µm), larger prokaryotes cover most of cell surface except peribuccal region occupied by smaller forms, two morphotypes not interspersed (Fig. 3M–O). Cells swim moderately fast (Supplementary video 1).

**Table 2:** Morphometric data for *Metacyclidium pallium* (strain AKAMAS2)

Characteristics^a^	Method	Mean	*M*	SD	CV	Min	Max	*n*
Cell length	in vivo	24.1	24.5	1.6	6.7	21	27	16
	*P*	19.2	19.2	1.3	6.9	16.3	22.1	31
Cell width	in vivo	10.6	10	1.0	9.8	9	12	16
	*P*	9.8	9.8	1.0	10.4	8	12.3	31
Cell length–width, ratio	in vivo	2.3	2.2	0.2	8.4	2	2.7	16
	*P*	2.0	2.0	0.1	7.6	1.7	2.3	31
Buccal region, length	in vivo	11.1	11	0.7	6.2	10	13	30
	*P*	9.3	9.3	0.4	4.6	8.3	10	22
Buccal region length/cell length %	in vivo	50	50	0.0	6.3	40	50	15
	*P*	50	50	0.0	5.3	40	50	21
Buccal region, width	in vivo	3.3	3	0.5	14.4	3	4	24
	*P*	2.2	2.1	0.3	12.6	2	2.8	11
Kinetids in mid-dorsal kinety, number	*P*	13.3	13	1.4	10.4	12	16	21
Kinetids in SK1, number	*P*	22.3	23	2.1	9.3	20	24	3
Somatic kineties, number	*P*	7.3	7.0	0.5	7.0	7	8	6
Transverse distance between mid-dorsal kineties	*P*	2.3	2.3	0.4	16.9	2	3.5	31
Distance between M3 and posterior end of the paroral	*P*	3.4	3.4	0.5	15.3	2.4	4.5	22
Somatic cilia, length	in vivo	5.4	5.5	0.4	7.7	5	6	8
Caudal cilium, length	in vivo	12.9	13	2.3	17.7	9	16	13
Macronucleus, diameter	*P*	4.1	4.1	0.3	8.0	3.7	4.9	15
Macronuclei, number	*P*	1.1	1.0	0.3	24.2	1	2	15
Micronucleus, diameter	*P*	1.5	1.5	0.1	7.2	1.3	1.6	22
Micronuclei, number	*P*	1.2	1.0	0.5	40.7	1	3	25
Ectosymbionts, cell length	SEM	2.8	2.9	0.5	18.1	2	3.9	24

^a^All distances in µm

*CV* coefficient of variation (%), *M* median, *M3* adoral membranelle 3, *Max* maximum value, *Mean* arithmetic mean, *Min* minimum value, *n* number of cells studied, *P* protargol, *SD* standard deviation of the arithmetic mean, *SEM* scanning electron microscopy, *SK1* somatic kinety 1

Somatic cilia short (about 5.5 µm long in vivo), arranged in seven or eight uninterrupted bipolar rows, kineties consist of dikinetids anteriorly and posteriorly with short intervening segment of monokinetids, 12–16 kinetids in mid-dorsal somatic kinety (Fig. 3B, C). Somatic kinety one (SK1) composed of 20–24 kinetids, anteriormost ones as dikinetids. Closely spaced kinetids of anterior part of SK1 parallel with and very close to paroral (Fig. 3B, E). Dikinetids in posterior cell part with only posterior basal body ciliated. Single caudal cilium, shorter than body length (Fig. 3A, G, I, M).

Buccal area extends about 50% of body length. Paroral reverse J-shaped anterior end reaches to anterior margin of adoral membranelle one (Fig. 3B, E, I, L; Supplementary Fig. S2 A–C). Adoral membranelle one (M1) consists of three longitudinal rows of basal bodies, only slightly subapical, M2 and M3 rhomboidal patches of basal bodies (Fig. 3E; Supplementary Fig. S2A, B). Scutica not identifiable in morphostatic cells. Cytopharyngeal basket not seen.

Morphogenesis follows typical scuticobuccokinetal pattern, opisthe oral structures arise from both posterior part of proter paroral and scutica. Parental paroral and adoral membranelles disaggregate and regenerate. Micronuclear karyokinesis completes before that of macronuclei. Somatic kineties arise from intrakinetal duplication of basal bodies (Fig. 3P; Supplementary Fig. S2E–G).

**Ecology and occurrence:** Marine habitat. Found in brackish and marine sediments from brackish coastal lakes and marine beach and tidal pool sediments. Found in Mediterranean Sea and Indian Ocean.

**Remarks:**

Only the AKAMAS2 population of *M. pallium* was studied in detail with silver impregnation methods.

***Maricyclidium*** **n. gen.**

**ZooBank registration:** urn:lsid:zoobank.org:act:B109538B-7A2D-4F3B-B156-FDDFB9EED677.

**Diagnosis:** With characters of the family. Paroral L-shaped. Somatic kineties interrupted, each consists of longer anterior fragment and a very short posterior fragment except somatic kinety 3 invariably lacks posterior fragment. Basal bodies absent in equatorial region. Scutica comprises a right and left pair of basal bodies just posterior to proximal part of paroral. Posterior fragment of somatic kinety n located more anteriorly than others, near left part of scutica. Prokaryotic ectosymbionts present.

**Type species:** *Maricyclidium commune* n. sp.

**Etymology:** Compound of *mari*- (marine) and *Cyclidium*, referring to the marine habitat of the *Cyclidium*-like genus. Neuter gender.

***Maricyclidium commune*** **n. sp.**

**ZooBank registration:** urn:lsid:zoobank.org:act:0B9A1327-3637-40E0-AC6F-5643EBAEFA06.

**Diagnosis:** Body size about 20 × 10 µm in vivo. Shape ovoidal to ellipsoidal, cells circular in cross section. Apical plate conspicuous, bulbous. Posterior ends of paroral cilia extend to or beyond posterior end of cell. Caudal cilium longer than body length. Eight interrupted longitudinal somatic kineties, anterior fragments comprise 7–11 kinetids, posterior fragments comprise 2 or 3 basal bodies. Contractile vacuole subterminal, excretory pore just behind posterior fragment of SK2. Cytopharyngeal basket not seen.

**Etymology:** From the Latin adjective *commune* (common), referring to the common presence of the species in various marine habitats.

**Type locality:** Marine beach sediment from Spiridonisos, Corfu, Greece (39°48′56.0″N 19°51′35.0″E).

**Type material:** One slide with the protargol-impregnated holotype and several paratypes of strain SIP1E is deposited in the collection of the National Museum of the Czech Republic, Prague, inventory number P6E 5598. The holotype is marked with a black ink circle and shown in Fig. 5.

**Gene sequence:** Partial 18S rRNA gene sequences from *Maricyclidium commune* n. sp., strains SIP1E, GUARD2, and SALT2, were deposited in GenBank with accession numbers PQ800185, PQ800186, and PQ800187, respectively.

**Fig. 4:**
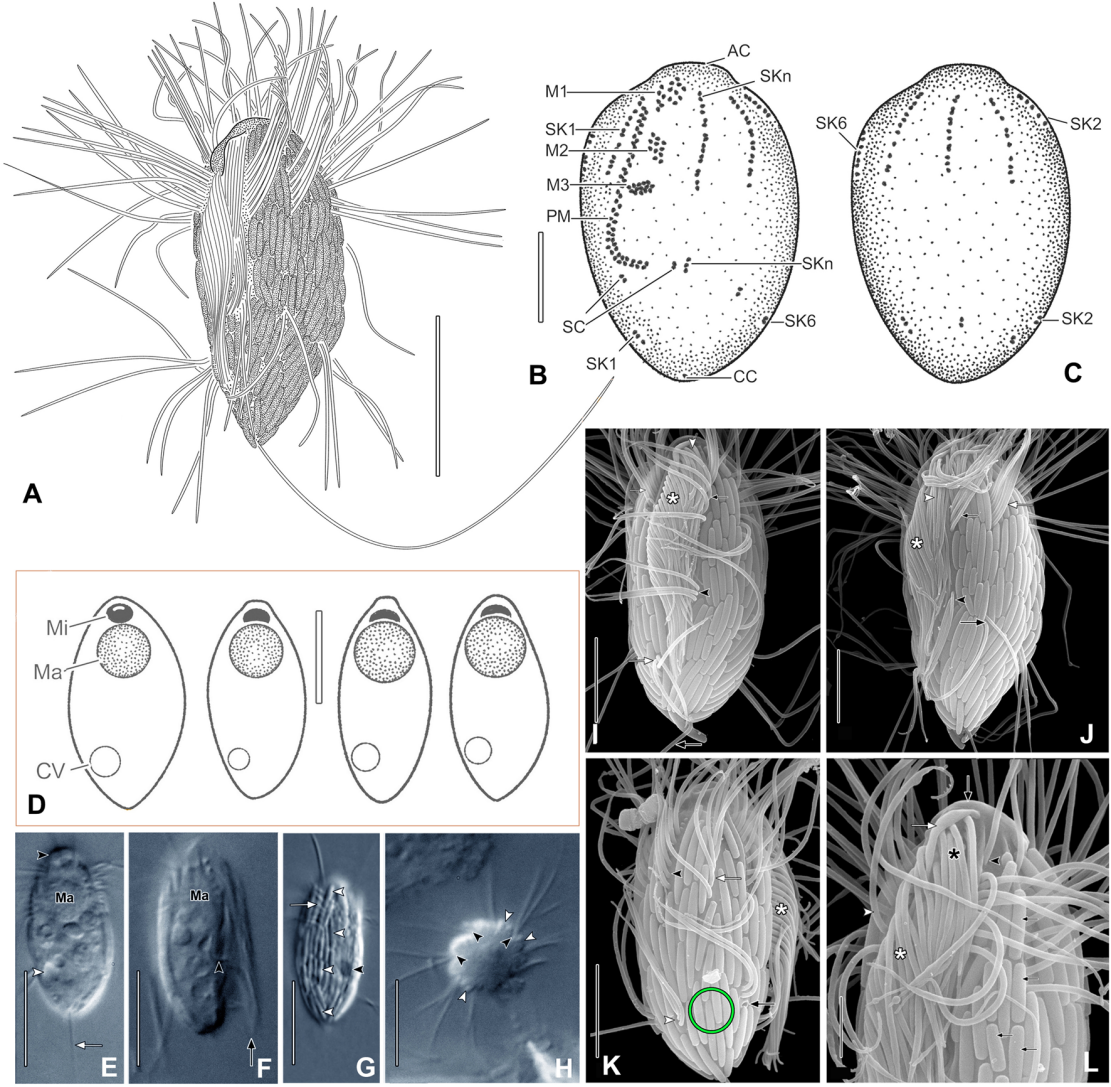
*Maricyclidium commune* n. gen., n. sp. from life (**A, D, E–H**), protargol (**B, C**), and in SEM (**I–L**). **A** Typical cell. **B, C** Infraciliature of holotype. **D** Nuclear apparatus. **E** View of AC (black arrowhead), CV (white arrowhead), and CC (arrow). **F** View of long PM cilia (arrow) and posterior end of PM (arrowhead). **G** View of symbionts (white arrowheads), SK fragment (arrow), and CV (black arrowhead). **H** View of SKs (black arrowheads) in furrows between symbionts (white arrowheads). **I** View of PM cilia (asterisk), SK1 (small white arrow, white arrow), SKn (small black arrow, black arrowhead), M1 (white arrowhead), and CC (black arrow). **J** View of PM (asterisk), M1 (white arrowhead), anterior SKn (small black arrow) and SKn–1 (white arrow), posterior SKn (black arrowhead) and SKn–1 (black arrow). **K** View of PM (asterisk), excretory pore at SK2 end (black arrow), SK3 (white arrow), and SK4 (arrowheads); SK3 lacks posterior fragment (green circle). **L** Detail of PM (white asterisk), M1 (black asterisk), AC (arrow), anterior SKn (arrowhead), and symbionts (small arrow). *AC* apical plate, *CC* caudal cilium, *CV* contractile vacuole, *Ma* macronucleus, *Mi* micronucleus, *M1–M3* adoral membranelles 1–3, *PM* paroral, *SK1* 2, 6, somatic kineties 1, 2, 6, *SKn* somatic kinety n. Scale bars: 10 µm (**A**, **D**, **E–H**); 5 µm (**B**, **C**, **I–L**)

**Description** (based on strain SIP1E; Fig. 4; Table 3; Supplementary Fig. S3, video 2)**:** Body size in vivo 18–21 × 9–11 µm, in protargol preparations, size 15–19 × 6–11 µm. Cells ovate to elliptical in outline, with prominent, bulbous apical plate (Fig. 4B, D, E). Single globular macronucleus in anterior one-third of cell (about 4.5 µm in diameter) with flattened peripheral nucleoli in protargol preparations. Single spherical micronucleus adjacent to macronucleus (about 1.5 µm in diameter) (Fig. 4D–F). Contractile vacuole subterminal, excretory pore at terminus of posterior fragment of SK2 (Fig. 4D, E, G, K). Cytoplasm colorless with scant food vacuoles (about 3–3.5 µm in diameter) and scattered globules (about 2 µm in diameter) (Fig. 4E, F). Extrusomes absent. Cells covered with longitudinally arranged fusiform ectosymbiotic prokaryotes (about 4.1 µm long) (Fig. 4A, G, K, L; Supplementary Fig. S3F). Swims rapidly, darting with intermittent brief pauses (Supplementary video 2).

**Table 3:** Morphometric data for *Maricyclidium commune* (strain SIP1E)

Characteristics^a^	Method	Mean	*M*	SD	CV	Min	Max	*n*
Cell length	in vivo	19.7	19.5	1.1	5.5	18.0	21.0	22
	*P*	16.1	15.8	1.4	8.7	13.3	18.9	20
Cell width	in vivo	10.0	10.0	0.7	6.6	9.0	11.0	22
	*P*	8.5	8.4	1.2	14.6	6.0	10.6	20
Cell length–width, ratio	in vivo	2.0	2.0	0.1	4.7	1.8	2.1	22
	*P*	1.9	1.9	0.2	8.0	1.6	2.2	20
Buccal region, length	in vivo	10.1	10.0	0.5	4.7	9.0	11.0	18
	*P*	9.1	9.0	0.6	6.5	8.1	10.2	16
Buccal region length/cell length %	in vivo	50	50	0.0	4.9	50	60	18
	*P*	60	60	0.0	6.3	50	60	16
Buccal region, width	*P*	1.8	2.0	0.4	21.2	1.2	2.1	5
Somatic kineties, number	CH-L	7.1	7.0	0.4	5.0	7.0	8.0	8
Kinetids in anterior part of somatic kinety, number	SEM	9.2	9.0	1.2	13.2	8.0	12.0	21
Kinetids in posterior part of somatic kinety, number	SEM	2.4	2.0	0.5	20.8	2.0	3.0	25
M1 to M3 distance	CH-L	5.6	5.6	0.5	8.8	5.1	6.7	11
Distance between M3 and posterior end of the paroral	*P*	3.5	3.5	0.2	6.5	3.3	4.0	13
Apical plate, width	in vivo	3.8	4.0	0.4	10.6	3.0	4.0	16
	*P*	3.7	3.6	0.5	14.6	2.8	4.6	13
Somatic cilia, length	in vivo	9.2	9.1	0.9	9.7	7.9	10.9	15
Caudal cilium, length	in vivo	17.6	17.0	1.4	7.8	16.0	20.0	12
Paroral cilia, length	in vivo	8.5	8.0	0.6	6.9	8.0	10.0	20
Macronucleus, diameter	*P*	3.3	3.1	0.5	16.1	2.8	4.9	20
Micronucleus, diameter	*P*	1.4	1.4	0.1	10.7	1.1	1.7	18
Ectosymbionts, cell length	in vivo	4.1	4.0	0.5	12.1	3.0	5.0	13
	SEM	3.6	3.6	0.6	16.7	2.2	4.9	31

^a^All distances in µm. Measurements made using ocular micrometer

*CH-L* Chatton–Lwoff silver nitrate method, *CV* coefficient of variation (%), *M* median, *M1–M3* adoral membranelle 1–3, *Max* maximum value, *Mean* arithmetic mean, *Min* minimum value, *n* number of cells studied, *P* protargol, *SD* standard deviation of the arithmetic mean, *SEM* scanning electron microscopy

Somatic cilia long (about 13 µm on average), arranged in eight equatorially interrupted longitudinal kineties. Anterior fragments of somatic kineties (SK) with 7–11 mixed mono- and dikinetids, posterior fragments comprise 2 or 3 kinetids (Fig. 4B, C, I, J). SK3 invariably lacks posterior fragment (Fig. 4I, J), posterior fragments of all other SKs except SKn subterminal in posterior one-quarter of cell; posterior fragment of SKn displaced anteriorly at level of posterior end of paroral, adjacent to left part of scutica (Fig. 4B; Supplementary Fig. S3). Anterior fragment of SK1 parallel with and very close to paroral (Fig. 4B, I; Supplementary Fig. S3 A, E). Single caudal cilium, usually longer than cell body (about 25 µm in SEM preparations) (Fig. 4A, E, I).

Paroral about 50% of body length, L-shaped, terminates anteriorly at posterior margin of adoral membranelle 1 (M1) (Fig. 4B, F, I, J; Supplementary Fig. S3A, C, D). Cilia of paroral membrane extend to form a conspicuous velum when cells at rest. M1 composed of about 9–12 irregularly arranged basal bodies, separated from anterior part of paroral by prominent cortical flap, M2 comprises roughly rectangular patch, sometimes several basal bodies between M1 and M2; M3 composed of three short, transversely oriented rows of basal bodies (often appears as two rows in protargol preparations, due to optical superimposition) (Fig. 4B; Supplementary Fig. S3). Scutica bipartite, left part consists of two basal bodies between paroral and posterior fragment of SKn, right part consists of two basal bodies to the right of posterior margin of paroral (Fig. 4B; Supplementary Fig. S3).

**Ecology and occurrence:** Marine habitat. Isolates from beach sediments of the Mediterranean Sea and salt marshes of the Atlantic coast of Rhode Island, USA.

**Remarks:**

**Fig. 5:**
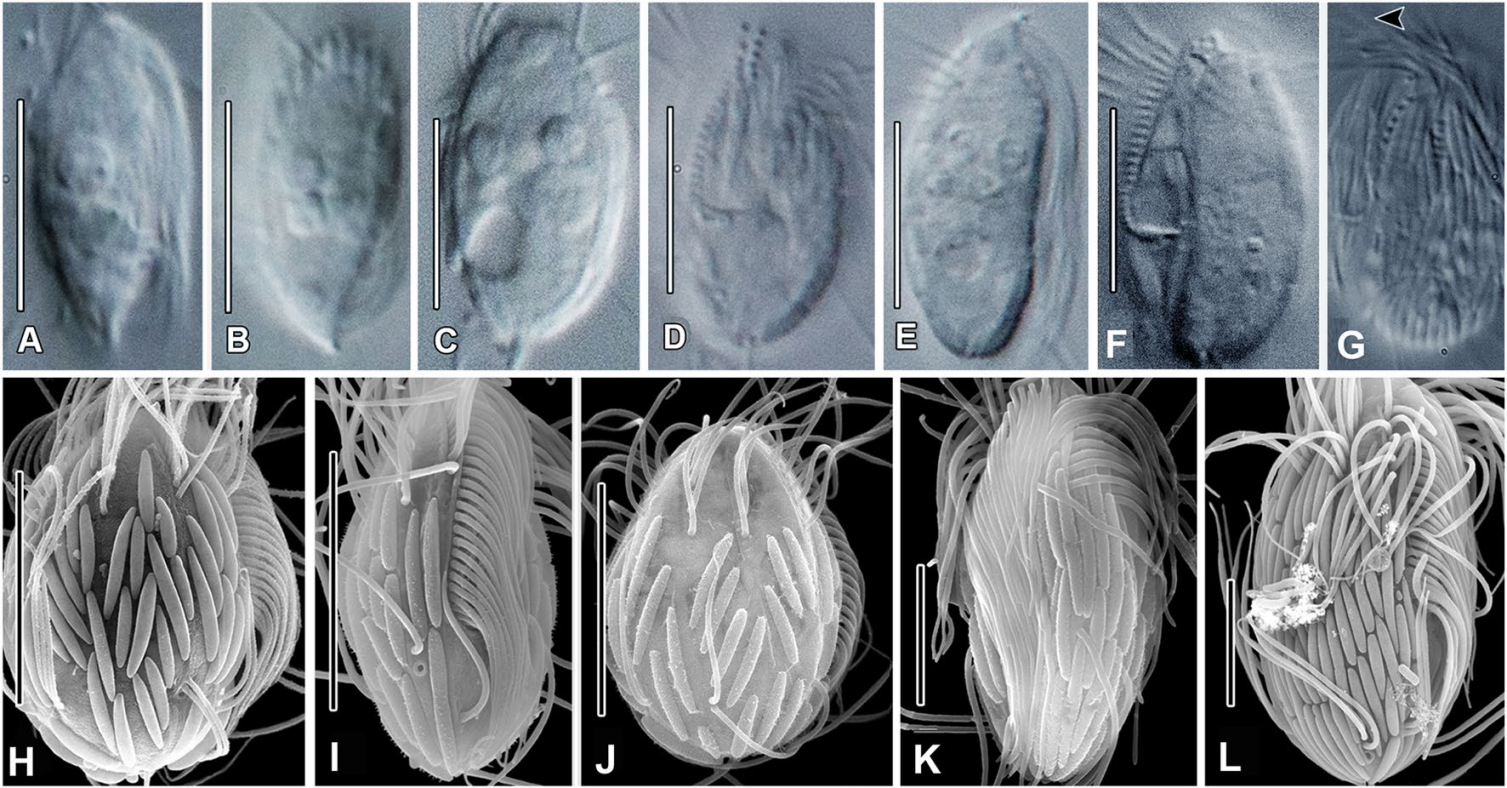
Undescribed *Maricyclidium* species: *Maricyclidium* lineage 1 (strain LJUBACKI, **A–C**, **I, J**), *M.* lineage 3 (strain OCE20C, **G**, **H**), *M.* lineage 2 (strain ALB1, **G, H**, **K**), and *M.* lineage 4 (strain CRVAR2B, **J**, **L**), in vivo (**A–G**), and in the scanning electron microscope (**H–L**). **A–C** Variations in cell shape. **D, E** Ventral (**D**) and right lateral (**E**) view. **F** Ventral view (**F**). **G** Right lateral view (G). **H** Right lateral view. **I, J** Right ventrolateral (**I**) and dorsal (**J**) views.** K** Left ventrolateral view. **L** Right ventrolateral view. Scale bars: 10 µm (**A–J**), 5 µm (**K**, **L**)

**Fig. 6:**
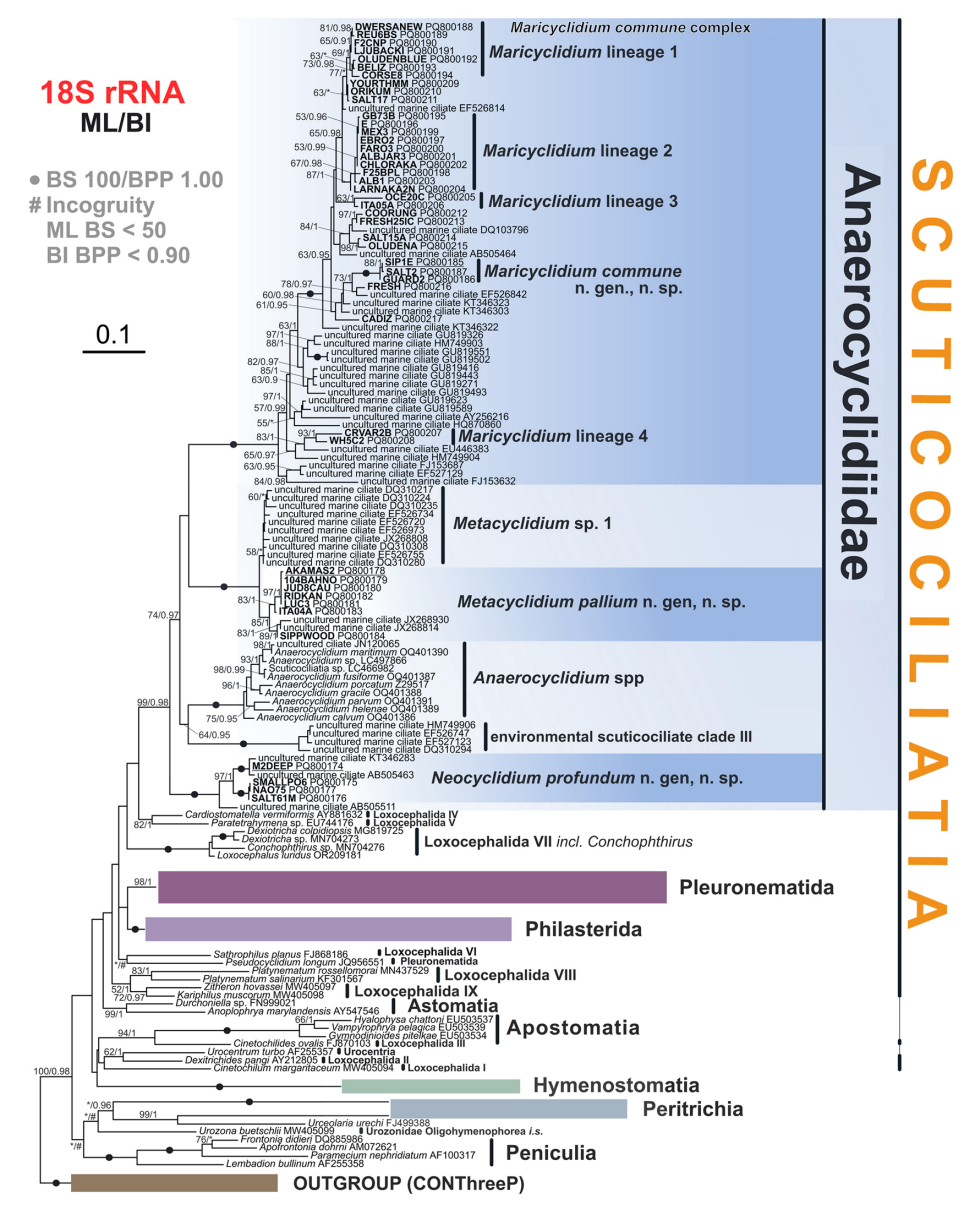
Phylogenetic tree of the class Oligohymenophorea based on 18S rRNA gene sequences showing newly described scuticociliate species. The tree was constructed by the maximum-likelihood method in RAxML under the GTR + Γ model. The tree is unrooted. The values at the branches represent statistical support in maximum-likelihood bootstrap values/Bayesian posterior probabilities. Fully supported branches (100/1) are marked with solid circles. Support values below 50/0.90 are not shown or depicted by an asterisk. Newly determined sequences are in bold. Type populations highlighted with an underscore. Environmental sequences (“uncultured” organisms) are represented by GenBank accession numbers. Scale bar: 10 nucleotide substitutions/100 base pairs

Four genetically closely related strains (see below) were studied in detail with silver impregnation methods and are referred to as undescribed *Maricyclidium* lineages 1–4. They show little morphologic variation from *M. commune* strain SIP1E and are, as yet, distinguishable from it and from each other only by their 18S rRNA gene sequences (Figs. 5, 6; Supplementary Fig. S4, Tables S2–S6, video 3). Some differences are noted, e.g., *Maricyclidium* lineage 1 (strain LJUBACKI) is significantly smaller than the others, *M.* lineage 4 (strain CRVAR2B) has a longer caudal cilium, a more posteriorly located excretory pore, and larger ectosymbionts. However, these differences are minor and, until more clear-cut distinguishing morphologic or ultrastructural features are identified, we refrain from formally identifying each of these strains as a new species.

***Maricyclidium*** **lineage 1 (strain LJUBACKI)**

**Locality:** Marine beach sediment in Adriatic Sea, Ljubač, Croatia (44°14′53.4"N 15°17′51.4"E).

**Voucher material:** One voucher slide of strain LJUBACKI with several protargol-impregnated specimens marked with black circles is deposited in the collection of the National Museum of the Czech Republic, Prague, inventory number P6E 5599.

**Gene sequence:** Partial 18S rRNA gene sequences from *Maricyclidium* lineage 1, strains LJUBACKI, DWERSANEW, REU6BS, F2CNP, OLUDENBLUE, BELIZ, and CORSE8, were deposited in GenBank with accession numbers PQ800191, PQ800188, PQ800189, PQ800189, PQ800190, PQ800192, PQ800193, and PQ800194, respectively.

***Maricyclidium*** **lineage 2 (strain ALB1)**

**Locality:** Sandy marine beach sediment in Orbetello, Italy (42°25′45.5"N 11°15′08.0"E).

**Voucher material:** One voucher slide of strain ALB1 with several protargol-impregnated specimens marked with black circles is deposited in the collection of the National Museum of the Czech Republic, Prague, inventory number P6E 5600.

**Gene sequence:** Partial 18S rRNA gene sequences from *Maricyclidium* lineage 2, strains ALB1, GB37B, E, EBRO2, F25BPL, MEX3, FARO3, ALBJAR3, CHLORAKA, and LARNAKA2N, were deposited in GenBank with accession numbers PQ800203, PQ800195, PQ800196, PQ800197, PQ800198, PQ800199, PQ800200, PQ800201, PQ800202, and PQ800204, respectively.

***Maricyclidium*** **lineage 3 (Strain OCE20C)**

**Locality:** Marine beach sediment in Indian Ocean, Mauritius (20°17′39.51"S, 57°47′20.31"E).

**Voucher material:** One voucher slide of strain OCE20C with several protargol-impregnated specimens marked with black circles is deposited in the collection of the National Museum of the Czech Republic, Prague, inventory number P6E 5601.

**Gene sequence:** Partial 18S rRNA gene sequences from *Maricyclidium* lineage 3, strains OCE20C and ITA05A, were deposited in GenBank with accession numbers PQ800205 and PQ800206, respectively.

***Maricyclidium*** **lineage 4 (strain CRVAR2B)**

**Locality:** Rocky beach sediment in Adriatic Sea, Červar, Croatia (45°15′51.5″N, 13°34′22.6″E).

**Voucher material:** One voucher slide of strain CRVAR2B with several protargol-impregnated specimens marked with black circles is deposited in the collection of the National Museum of the Czech Republic, Prague, inventory number P6E 5602.

**Gene sequence:** Partial 18S rRNA gene sequences from *Maricyclidium* lineage 4, strains CRVAR2B and WH5C2, were deposited in GenBank with accession numbers PQ800207 and PQ800208, respectively.

### 18S rRNA gene phylogeny

Our phylogenetic analyses (Fig. 6), based on partial 18S rRNA gene sequences, are consistent with previous studies on scuticociliates (Feng et al. 2015; Gao et al. 2012a; Liu et al. 2025; Poláková et al. 2021, 2023; Zhang et al. 2019). The subclass Scuticociliatia comprises orders Philasterida, Pleuronematida, and Loxocephalida, along with the recently erected family Anaerocyclidiidae, with philasterids being the only monophyletic order. The individual lineages of the polyphyletic Loxocephalida are labeled according to Poláková et al. (2021). We obtained 44 partial 18S rRNA gene sequences of scuticociliates isolated from marine anoxic habitats. All newly determined sequences, together with 42 environmental sequences from marine anoxic habitats, obtained from GenBank, represent the environmental scuticociliate clades I–IV sensu Poláková et al. (2023) and, together with the genus *Anaerocyclidium*, form a robust deep-branching clade of Scuticociliatia (bootstrap support [BS] 99/Bayesian posterior probability [BPP] 0.98). Although our single-gene phylogeny could not resolve the exact phylogenetic position of this clade within the subclass Scuticociliatia, it remains phylogenetically distinct from orders Pleuronematida and Philasterida. As a result, we expand the family Anaerocyclidiidae Poláková et al. 2023 to include all of the marine environmental scuticociliate clades I–IV related to the predominantly freshwater genus *Anaerocyclidium*. We erect three new genera for the environmental scuticociliate clades I, II, and IV, designated as *Neocyclidium* n. gen., *Metacyclidium* n. gen, and *Maricyclidium* n. gen., respectively. *Neocyclidium profundum* n. gen., n. sp. is represented by four strains, two of which were isolated from deep-water sediments, as well as by three environmental sequences from GenBank, all originating from deep-water habitats. *Metacyclidium pallium* n. gen., n. sp. is represented by seven strains and two environmental sequences from GenBank. Several other environmental sequences form a lineage closely related to *M. pallium* but since there is no morphological data for them, we refer to them as *Metacyclidium* sp. 1. *Maricyclidium commune* n. gen., n. sp. belongs to the most diversified clade of the marine Anaerocyclidiidae (i.e., environmental scuticociliate clade IV sensu Poláková et al. 2023). Since the lack of clear distinguishing morphological features prevents us from formally describing them as new species, we refer to them here as *Maricyclidium* lineages 1–4. Until further evidence is available, we consider the entire clade as the *M. commune* species complex. Genetic distances (uncorrected p-distances) between the individual *Maricyclidium* lineages ranged from 0.01 to 0.068 (Supplementary Table S7). Most *Maricyclidium* lineages exhibit little to no intraspecific variability (maximum genetic distance of 0.008), with only *Maricyclidium* lineage 4 showing higher variability (genetic distance up to 0.032). Environmental scuticociliate clade III remains the only genus-level lineage of Anaerocyclidiidae with no morphological data available.

Consequently, the family Anaerocyclidiidae now comprises five fully supported clades of marine and freshwater anaerobic scuticociliates represented by four genera and nine species. The newly determined 18S rRNA gene sequences have been deposited in GenBank database under accession numbers PQ800174–PQ800217.

### Symbionts and cell ultrastructure

**Fig. 7:**
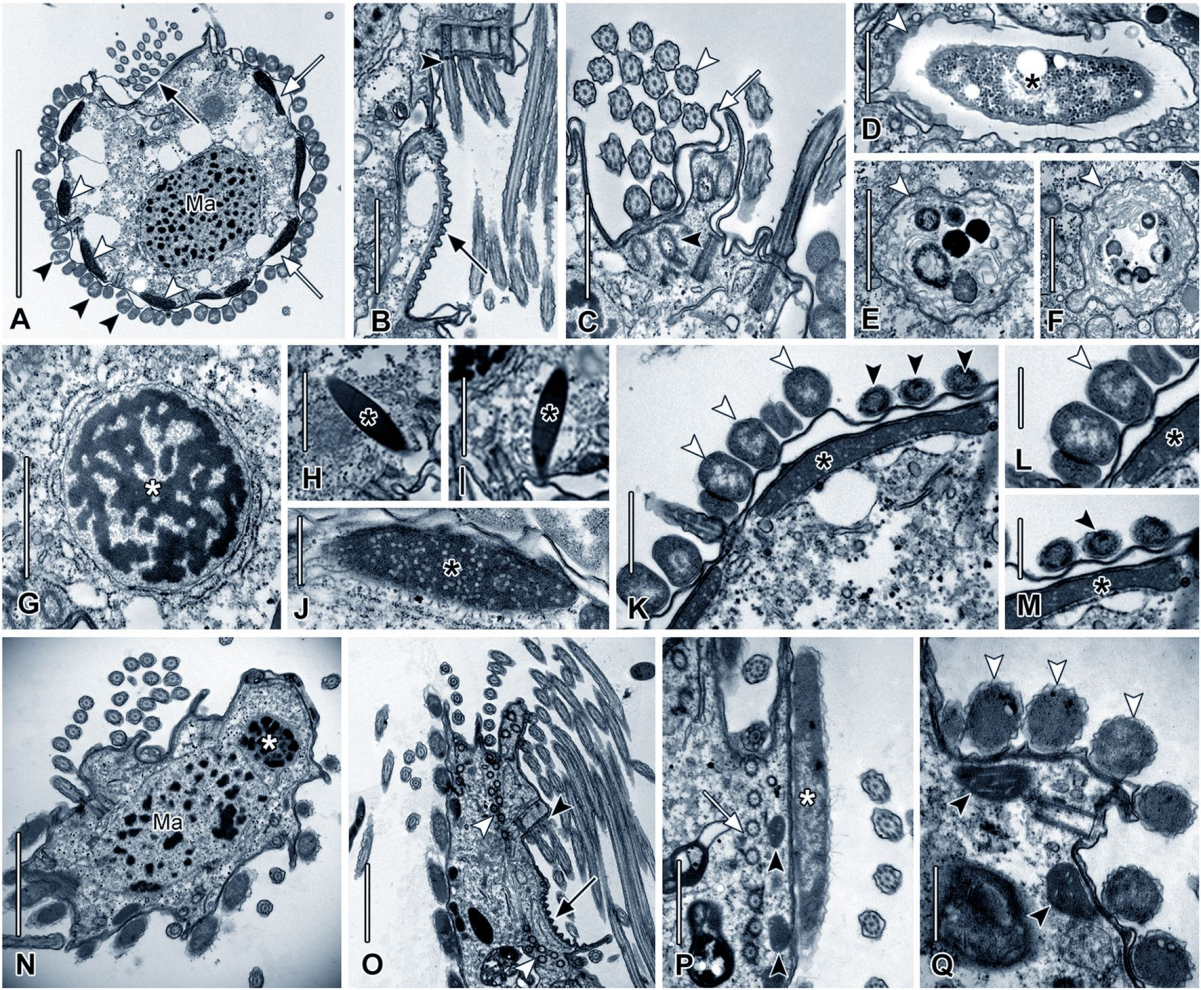
Ultrastructure of *Metacyclidium pallium* n. gen., n. sp. strain AKAMAS2 (**A–M**), *Maricyclidium* lineage 1 strain LJUBACKI (**N–P**), and *M*. lineage 4 strain CRVAR2B (**Q**), in TEM. **A** Section of oral apparatus (black arrow), cortical alveoli (white arrow), mitochondria (white arrowhead), and symbionts (black arrowheads). **B** Detail of oral ribs (black arrow) and cilia of an adoral membranelle (black arrowhead). **C** Section of oral cilium (white arrowhead), dikinetid (black arrowhead), and cortical alveolus (white arrow). **D–F** Food vacuoles (white arrowheads) and engulfed prokaryote (asterisk).** G** Micronucleus. **H, I** Extrusomes. **J** Mitochondrion (asterisk) with tubular cristae. **K** Association of mitochondria (black asterisks) with large fusiform (white arrowheads) and slender cylindroidal (black arrowheads) symbionts. **L, M** Large (white arrowhead) and slender (black arrowhead) symbionts. **N** Ma with attached micronucleus (white asterisk). **O** Oral ribs (black arrow), base of adoral membranelle (black arrowhead), and paroral dikinetids (white arrowheads). **P** Mitochondria (arrowheads) near symbiont (asterisk) and somatic dikinetid (arrow). **Q** Cortical mitochondria (black arrowheads) associated with symbionts (white arrowheads). Ma, macronucleus. Scale bars: 5 µm (**A**); 2 µm (**B**, **E**, **F**, **O**); 1 µm (**C**, **D**, **G**, **K**, **P**); 500 nm (**H–J**, **L**, **M**, Q)

We studied the ultrastructure of three strains: one of *Metacyclidium pallium* (strain AKAMAS2) and two from the *Maricyclidium commune* species complex, *Maricyclidium* lineage 1 (strain LJUBACKI) and *M.* lineage 4 (strain CRVAR2B). Typical ciliate features were documented, including the macronucleus, micronucleus, and structures of the somatic and oral ciliature (Fig. 7A–C, G, N–P). In vivo imaging and transmission electron microscopy (TEM) show sparsely distributed fusiform extrusomes in *M. pallium* (Figs. 3K, L, 7H, I). Thus,* M*. *pallium* is, as yet, the only member of Anaerocyclidiidae shown to have extrusomes (see Poláková et al. 2023). Cells of *M. pallium* also contained numerous food vacuoles containing bacterial prey, confirming their bacterivory (Fig. 7D–F). Cytoplasmic endosymbionts were not seen in vivo or in silver impregnations and were absent in transmission electron photomicrographs. In contrast to the predominantly freshwater Anaerocyclidiidae which possess endosymbionts (Poláková et al. 2023), the marine strains in the current study displayed rod-shaped to fusiform, longitudinally oriented prokaryotic ectosymbionts, visible in vivo, in silver-impregnated specimens, and in scanning electron photomicrographs (Figs. 2E, I, 3O, N, 4G, K, 5H–L, O, 7K–M, P, Q). Although varying between strains, each strain, itself, except one, had ectosymbionts of a single morphotype. The one exception was *Metacyclidium pallium* which had two distinct ectosymbiont morphotypes (one large, fusiform and the other small, rod-shaped) (Figs. 3A, M, O, 7K–M). The mitochondria of the three strains studied by TEM possessed tubular cristae (Fig. 7J, Q), and were located in the cell cortex near the symbionts. While a similar mitochondrial distribution has been observed in other scuticociliates lacking ectosymbionts, the cortical positioning of mitochondria may also be a prerequisite for ectosymbiont attachment and metabolic interaction with the host mitochondria (Fig. 7K–M, P, Q) (e.g., de Castro et al. 2014; Paramá et al. 2006).

## Discussion

### Capturing environmental diversity through cultivation

Scuticociliates represent a highly diverse lineage of ciliates that, in addition to aerobic biotopes, have successfully colonized a wide range of microoxic-to-anoxic marine habitats, often being abundant in these environments (Coyne et al. 2013; Edgcomb and Pachiadaki 2014; Hu et al. 2024; Orsi et al. 2012a, b; Wylezich and Jürgens 2011; Zhao et al. 2017). Despite their ecological significance, until now, little was known about the morphology and behavior of anaerobic scuticociliates.

In this study, we provide detailed morphological and molecular evidence confirming the frequent occurrence of scuticociliates in oxygen-depleted marine habitats. Additionally, we describe three new genera that encompass most of the environmental lineages identified to date. Only a single lineage of Anaerocyclidiidae, here referred to as environmental scuticociliate clade III, was not captured by our sampling and cultivation efforts and awaits further investigation.

Our findings also demonstrate that anaerobic scuticociliates have a broad, likely global, geographic distribution, and occur in both littoral and deep-water sediments. Notably, *Neocyclidium profundum* n. sp. was predominantly found in deep-water ecosystems, as evidenced by both our cultivation efforts and environmental surveys (Pasulka et al. 2016; Takishita et al. 2010), indicating its adaptation to the extreme conditions characteristic of these habitats. The cultivation of deep-water isolates of *N. profundum* represents one of the rare successful efforts in cultivating deep-water ciliates (Schoenle et al. 2017, 2021; Živaljić et al. 2020).

Importantly, the establishment of stable laboratory cultures of anaerobic scuticociliates enabled us to confirm their anaerobic lifestyle, morphologies, and symbiotic partnerships. This underscores the value of cultivation as a powerful tool for capturing the diversity and understanding the ecology of this anaerobic lineage.

### Comparison of new genera with other described species

The only anaerobic *Cyclidium*-like ciliate previously documented from marine habitats is *Cyclidium xenium* Fenchel et al., 1995. Although it shares a similar arrangement of kinetids with *Neocyclidium* n. gen., it differs significantly in buccal area length (about 80% of the body length vs. about 50%) and the absence of ectosymbiotic prokaryotes. *Maricyclidium* n. gen. can be clearly distinguished from the other Anaerocyclidiidae genera (*Anaerocyclidium*, *Metacyclidium*, and *Neocyclidium*) by the presence of interrupted somatic kineties. The two other newly described marine genera of Anaerocyclidiidae, *Neocyclidium* n. gen. and *Metacyclidium* n. gen., share the presence of uninterrupted bipolar kineties. However, *Metacyclidium* can be clearly distinguished from *Neocyclidium* by the cell shape (more rounded anteriorly vs. more narrowed anteriorly), extrusomes (present vs. absent), conspicuous cortical furrows (present vs. absent), contractile vacuole position (subterminal vs. terminal), and ectosymbionts (two distinct morphotypes vs. only one). Importantly, the close relationship between *Neocyclidium* and *Metacyclidium* is strongly rejected by the approximately unbiased test of constrained trees (*P* = 3.27e-11) (Supplementary Table S8).

### *Maricyclidium* as a new genus

The main distinguishing feature of the genus *Maricyclidium* is the presence of interrupted somatic kineties (i.e., the presence of a distinct equatorial area without basal bodies). Several other free-living scuticociliates with *Cyclidium*-like morphologies have interrupted somatic kineties, namely, *Cyclidium litomesum* Stokes, 1884, *C. heptatrichum* Schewiakoff, 1893, *C. libellus* Kahl, 1926, *C. oligotrichum* Kahl, 1928, *Gymnocyclidium nabranicum* Alekperov, 2009, *Paurotricha cyclidiformis* Dragesco and Dragesco-Kernéis, 1991, and *Isocyclidium globosum* Esteban and Finlay 1994. However, all of the aforementioned *Cyclidium*-like species were isolated from freshwater habitats and, except for *Isocyclidium globosum*, all lack ectosymbiotic prokaryotes, a feature characteristic of the marine *Maricyclidium commune* and *M.* lineages 1–4. *Gymnocyclidium* further differs from *Maricyclidium* by somatic ciliature (basal bodies arranged in three parts vs. two), shape of paroral (distinctly J-shaped vs. L-shaped), morphology of adoral membranelles 1 and 2 (M1 as two longitudinal rows vs. as large patch of basal bodies and M2 triangular vs. a rhomboidal collection of basal bodies), scutica (as single pair of basal bodies vs. bipartite), and number of caudal cilia (two vs. one). Although harboring ectosymbionts, *Isocyclidium globosum* can be clearly distinguished from *Maricyclidium* by habitat (freshwater vs. marine), body size (body length 20–45 µm vs. maximum body length 22.8 µm), shape (almost spherical vs. ellipsoidal), six basal bodies on kinety posterior fragments (vs. three or fewer), and methanogenic endosymbionts (present vs. absent).

### *Maricyclidium commune* species complex

Although a variety of methods were applied to examine the morphology of studied *Maricyclidium* strains, including in vivo imaging, silver impregnation techniques, and electron microscopy, the lack of sufficiently distinctive features in the *Maricyclidium* lineages prevents clear species delimitation, although, according to genetic distances (Supplementary Table S7), it is likely that *Maricyclidium* comprises more species. While these lineages might be considered as cryptic species by some, we refrain from using this designation due to ambiguities in defining crypticity (e.g., Fišer et al. 2018). Thus, we consider only strains SIP1E, SALT2, and GUARD2 to represent *Maricyclidium commune* n. sp. We provide characterizations for four other, morphologically very similar, lineages of the *M. commune* species complex, referred to here as *Maricyclidium* lineages 1–4 (Fig. 5; Supplementary Fig. S4, Tables S2–S6). All studied strains share similar overall morphology, having ellipsoidal cells with a truncate apical plate, a reverse J-shaped paroral with very long cilia, and interrupted somatic kineties forming tufts of cilia, longer in the anterior part and much shorter in the posterior part of the cell. Body size varies somewhat among *Maricyclidium* lineages with *M. commune* being the smallest (body length 15–19 µm in vivo) and *M.* lineage 4 being the largest (body length 17.2–22.8 µm in vivo), but, since body size varies with many factors (e.g., environmental conditions, nutrition state, stage in the cell cycle), it is usually, by itself, not a reliable or stable taxonomic character in *Cyclidium*-like scuticociliates. Notably, *Maricyclidium* lineage 4 strain CRVAR2B harbors significantly larger ectosymbionts compared to the other lineages (6.4 µm long rods vs. 4.1–4.7 µm in other lineages, on average).

### An independent origin of scuticociliate anaerobiosis in Oligohymenophorea

Although our single-gene phylogenetic analyses did not resolve the internal relationships among scuticociliate orders, our findings suggest that the family Anaerocyclidiidae represents an independent evolutionary origin of anaerobiosis within the class Oligohymenophorea. Despite possessing enzymes typical of anaerobes, the relatively limited reduction of aerobic enzyme complexes in *Anaerocyclidium porcatum*, along with the presence of mitochondrial cristae in Anaerocyclidiidae, further indicates a relatively recent transition from an aerobic ancestor to anaerobiosis (Lewis et al. 2020; Poláková et al. 2023; Fig. 7J, Q). Notably, the enhanced metabolic capacity of Anaerocyclidiidae may confer a significant advantage, allowing them to better tolerate oxygen fluctuations compared to obligate anaerobes with highly reduced metabolic systems. This adaptability is highlighted by the presence of anaerobic scuticociliates across a range of environments, from the oxic–anoxic interface to habitats without measurable oxygen (e.g., Coyne et al. 2013; Edgcomb et al. 2011a; Pasulka et al. 2016; Takishita et al. 2010; Wylezich and Jürgens 2011).

### Symbionts of Anaerocyclidiidae may be shaped by the environment

Mitochondrial metabolism typically undergoes extensive modifications during the adaptation to low- or zero-oxygen levels in the environment (e.g., Leger et al. 2019; Stairs et al. 2015). Although we have no data yet about the energetic metabolism of marine anaerobic scuticociliates, *Anaerocyclidium porcatum*, the only anaerobic scuticociliate whose mitochondrial metabolism has been studied in detail, possesses a hydrogen-producing mitochondrion-related organelle with cristae (Lewis et al. 2020). It is likely that the mitochondria of other members of Anaerocyclidiidae also produce hydrogen, which could serve as a substrate for prokaryotic symbionts forming syntrophic relationships with their ciliate hosts. While freshwater Anaerocyclidiidae harbor methanogenic symbionts inside the cells (endosymbionts) and have mitochondria distributed throughout the cytoplasm, all of the studied marine Anaerocyclidiidae exhibit a different arrangement of symbionts and mitochondria—the cells lack endosymbionts and are, instead, covered with ectosymbionts associated with host subcortical mitochondria (Esteban et al. 1993; Poláková et al. 2023; Fig. 7K–M, P, Q). While a similar mitochondrial distribution has been observed in other scuticociliates lacking ectosymbionts, the cortical positioning of mitochondria is also known from other ectosymbiont-bearing anaerobic protists (de Castro et al. 2014; Edgcomb et al. 2011c; Paramá et al. 2006). This arrangement of mitochondria may either serve as prerequisite for the ectosymbiont attachment and close metabolic interaction with host mitochondria or a consequence of the symbiosis. Although symbioses in marine environments remain understudied, one example of hydrogen-scavenging prokaryotes capable of forming syntrophic interactions with marine anaerobic ciliates are sulfate-reducing deltaproteobacteria (Beinart et al. 2018; Edgcomb et al. 2011b; Fenchel and Ramsing 1992; Orsi et al. 2012a, b; Rotterová et al. 2020). Similar associations may also occur in marine Anaerocyclidiidae, as they are frequently found in sulfidic sediments (Behnke et al. 2006, 2010; Stoeck et al. 2009; Wylezich and Jürgens 2011). Overall, habitat and environmental conditions (e.g., sulfide concentrations) are likely important factors influencing the identity of symbionts in anaerobic scuticociliates and, consequently, the mode of symbiosis (intracellular vs. extracellular localization) (Epstein et al. 1998; Esteban and Finlay 1994; Fenchel and Finlay 1991; Radek 2010). Distinct symbiont morphologies in the newly described species suggest a degree of specificity of host–symbiont interactions, as demonstrated in metopid and plagiopylid ciliates; however, this awaits further investigation (Méndez-Sánchez et al. 2024; Schrecengost et al. 2024). Notably, the discovery of ectosymbionts of varying sizes and shapes in the newly described Anaerocyclidiidae species, including the identification of two distinct morphotypes on the cortex of *Metacyclidium pallium* cells (Figs. 3M–O, 7K–M), underscores the likelihood of unexplored metabolic diversity in the symbioses in anaerobic scuticociliates.

## Conclusions

In this study, we provide detailed morphological and molecular characterization of three new genera and three new species of the family Anaerocyclidiidae isolated from marine anoxic habitats. We demonstrated that cultivation is a powerful tool for studying protist diversity in marine habitats, particularly for frequently occurring and abundant lineages such as anaerobic scuticociliates. As bacterivores with high-grazing rates, scuticociliates likely have a significant impact on shaping prokaryotic communities also in anoxic sediments (Hu et al. 2021; Posch et al. 2001; Šimek et al. 1996). We successfully established cultures of marine Anaerocyclidiidae, including deep-water representatives, providing a valuable resource for future research on this ecologically important ciliate lineage. We also showed that 18 S rRNA gene is a suitable marker for resolving diversity within Anaerocyclidiidae. However, multigene phylogenetic or phylogenomic approaches are needed to fully resolve the relationships among scuticociliates. Nevertheless, Anaerocyclidiidae represent an independent evolutionary lineage of scuticociliates.

## Supplementary Information

Below is the link to the electronic supplementary material.

Supplementary file1 (PDF 560 KB)

Supplementary file2 (CSV 4 KB)

Supplementary file3 (PDF 1900 KB)

Supplementary file4 (MP4 8165 KB)

Supplementary file5 (MP4 11841 KB)

Supplementary file6 (MP4 10196 KB)

## Data Availability

All data supporting the findings of this study are available within the article and its supplementary materials. DNA sequence data have been deposited in GenBank under accession numbers PQ800174–PQ800217. Additional data are available from the corresponding author upon request.
