# Supplementary material for: Capturing the extensive diversity of marine anaerobic scuticociliates (Oligohymenophorea, Ciliophora) through cultivation
Source: Mar Life Sci Technol. 2026 Mar 30;8(2):256–75. doi: 10.1007/s42995-025-00350-5 (PMC13198587; doi:10.1007/s42995-025-00350-5)
Supplement: Supplementary file 1 — Supplementary file1 (PDF 560 KB) [file 42995_2025_350_MOESM1_ESM.pdf]

Supplementary Table S1. New isolates presented in this study with available 18S rRNA gene sequences.

Strain	Species	Locality	Coordinates	Habitat type	Sample type	Date isolated
**104BAHNO**	*Metacyclidium pallium*	Hyères, France	43°03'49.2"N 6°08'50.9"E	brackish	sediment	2017
**AKAMAS2**	*Metacyclidium pallium*	South Akamas beach, Cyprus	35°02'27.8"N 32°16'36.4"E	marine	tidal pool sediment	2018
**ALB1**	*Maricyclidium* lineage 2	Orbetello, Italy	42°25'45.5"N 11°15'08.0"E	marine	sandy beach sediment	2017
**ALBJAR3**	*Maricyclidium* lineage 2	Knysna, South Africa	34°02'40.1"S 23°02'52.5"E	marine	sediment	2014
**BELIZ**	*Maricyclidium* lineage 1	Caye Caulker, Beliz	17°43'58.9"N 88°01'41.2"W	marine	coastal mangrove sediment	2014
**CADIZ**	*Maricyclidium commune* complex	Cádiz, Spain	36°36'04.9"N 6°16'41.3"W	marine	sandy beach sediment	2015
**CHLORAKA**	*Maricyclidium* lineage 2	Chloraka, Cyprus	34°47'35.0"N 32°23'39.0"E	marine	tidal pool sediment	2018
**COORUNG**	*Maricyclidium commune* complex	Salt Creek, South Australia	36°04'00"S 139°35'00"E	marine	sediment	2014
**CORSE8**	*Maricyclidium* lineage 1	Corsica, France	42°39'32.3"N 9°03'35.2"E	marine	marine river estuary	2017
**CRVAR2B**	*Maricyclidium* lineage 4	Crvar, Croatia	45°15'53.4"N 13°34'21.9"E	marine	rocky beach sediment	2014
**DWERSANEW**	*Maricyclidium* lineage 1	Eastern Cape province, RSA	32°17'34.4"S 28°49'51.4"E	marine	tidal pool area	2014
**E**	*Maricyclidium* lineage 2	Cyprus	n.a.	marine	beach sediment	2020

**EBRO2**	*Maricyclidium* lineage 2	Ebro, Spain	17°43'58.9"N 88°01'41.2"W	brackish	marine estuary outlet	2013
**F25BPL**	*Maricyclidium* lineage 2	Sri Lanka	8°34'27.1"N 81°11'36.2"E	marine	mangrove sediment	2012
**F2CNP**	*Maricyclidium* lineage 1	Sri Lanka	8°34'27.1"N 81°11'36.2"E	marine	mangrove sediment	2012
**FARO3**	*Maricyclidium* lineage 2	Ria Formosa, Olhão, Portugal	37°01'49.4"N 7°48'32.0"W	marine	saltmarsh sediment	2018
**FRESH**	*Maricyclidium commune* complex	Siders Pond, Falmouth, MA, USA	41°32'30.8"N 70°37'19.9"W	marine	coastal marsh salt pond outlet sediment	2016
**FRESH25IC**	*Maricyclidium commune* complex	Siders Pond, Falmouth, MA, USA	41°32'30.8"N 70°37'19.9"W	marine	coastal marsh salt pond outlet sediment	2019
**GB73B**	*Maricyclidium* lineage 2	Cyprus	n.a.	marine	beach sediment	2020
**GUARD2**	*Maricyclidium commune*	Nauset Bay, Eastham, USA	41°50'03.1"N 69°57'00.0"W	marine	beach sediment	2019
**ITA04A**	*Metacyclidium pallium*	Marinello, Sicily, Italy	38°08'13.7"N 15°03'13.7"E	brackish	coastal lake	2021
**ITA05A**	*Maricyclidium* lineage 3	Marinello, Sicily, Italy	38°08'33.1"N 15°03'00.0"E	marine	beach sediment	2021
**JUD8CAU**	*Metacyclidium pallium*	Point Judith Pond, Narragansett, RI, USA	41°22'50.2"N 71°30'01.4"W	marine	salt marsh sediment	2022
**LARNAKA2AN**	*Maricyclidium* lineage 2	Larnaka, Cyprus	34°51'00.0"N 33°37'00.0"E	marine	salt marsh sediment	2018
**LJUBACKI**	*Maricyclidium* lineage 1	Ljubač, Croatia	44°14'53.4"N 15°17'51.4"E	marine	beach sediment	2014
**LUC3**	*Metacyclidium pallium*	Brač island, Croatia	43°17'03.9"N 16°52'00.9"E	marine	sea sediment (several meters	2012

					deep)	
**M2DEEP**	*Neocyclidium profundum*	Santa Barbara Basin, CA, USA	34°18'07.0"N 120°00'56.1"W	marine	deep-water sediment (at 556 meters depth)	2022
**MEX3**	*Maricyclidium* lineage 2	Yucatán, Mexico	21°23'26.1"N 88°53'31.4"W	marine	coastal marsh	2018
**NAO75**	*Neocyclidium profundum*	CA, USA	33°38'24.0"N 118°48'01.3"W	marine	ocean bottom deep-water sediment (at 899 meters depth)	2016
**OCE20C**	*Maricyclidium* lineage 3	Mauritius	20°17'39.51"S 57°47'20.31"E	marine	beach sediment	2013
**OLUDENA**	*Maricyclidium commune* complex	Ölüdeniz, Turkey	36°32'33.8"N 29°07'31.0"E	marine	sandy beach sediment	2019
**OLUDENBLUE**	*Maricyclidium* lineage 1	Ölüdeniz, Turkey	36°32'33.8"N 29°07'31.0"E	marine	sandy beach sediment	2019
**ORIKUM**	*Maricyclidium commune* complex	Orikum, Albania	40°20'25.0"N 19°28'11.6"E	marine	sandy beach sediment	2019
**REU6BS**	*Maricyclidium* lineage 1	Réunion, France	20°52'30.8"S 55°26'38.8"E	brackish	beach sediment	2016
**RIDKAN**	*Metacyclidium pallium*	Bahia honda State Park, USA	24°39'35.5"N 81°16'23.8"W	marine	sandy beach sediment	2015
**SALT15A**	*Maricyclidium commune* complex	Salt Pond, Faltmouth, MA, USA	41°32'23.9"N 70°37'49.4"W	marine	salt marsh sediment	2019
**SALT17**	*Maricyclidium commune* complex	Salt Pond, Faltmouth, MA, USA	41°32'23.9"N 70°37'49.4"W	marine	salt marsh sediment	2019
**SALT2**	*Maricyclidium commune*	Salt Pond, Faltmouth, MA, USA	41°32'23.9"N 70°37'49.4"W	marine	salt marsh sediment	2016
**SALT61M**	*Neocyclidium profundum*	Salt Pond, Faltmouth, MA, USA	41°32'23.9"N 70°37'49.4"W	marine	salt marsh sediment	2016
**SIP1E**	*Maricyclidium commune*	Spiridonisos, Corfu, Greece	39°48'56.0"N 19°51'35.0"E	marine	beach sediment	2013

**SIPWOOD**	*Metacyclidium pallium*	Little Sippewissett Marsh, Faltmouth, MA, USA	41°34'33.1"N 70°38'25.7"W	brackish	salt marsh sediment	2016
**SMALLPO6**	*Neocyclidium profundum*	Little Pond, Falmouth, MA, USA	41°33'11.3"N 70°35'21.8"W	brackish	sediment from a shallow lake covered with kelp	2016
**WH5C2**	*Maricyclidium* lineage 4	Salt Pond, Faltmouth, MA, USA	41°32'24.1"N 70°37'49.4"W	marine	salt marsh sediment	2016
**YOURTHMM**	*Maricyclidium commune* complex	Hyannis, Cape Cod, MA, USA	41°38'09.5"N 70°16'52.6"W	marine	salt marsh pond sediment	2016

n.a., unknown.

Supplementary Table S2. Morphometric data for *Maricyclidium* lineage 1 (strain LJUBACKI).

Characteristic[a]	Method	Mean	M	SD	CV	Min	Max	*n*
Cell length	in vivo	18.3	17.7	1.3	7.0	16.7	20.9	20
	P	13.6	13.9	1.7	12.8	7.7	16.8	31
Cell width	in vivo	10.6	10.6	0.7	6.8	8.6	11.7	20
	P	8.5	8.6	1.0	11.3	5.7	10.1	31
Cell length-width, ratio	in vivo	1.7	1.8	0.1	6.5	1.5	1.9	20
	P	1.6	1.6	0.2	12.0	1.0	2.1	31
Buccal region, length	P	8.3	8.2	0.7	8.8	7.1	10.0	18
Buccal region length/cell length %	P	60	60	0.1	11.2	50	70	18
Buccal region, width	P	1.7	1.8	0.2	13.9	1.4	1.8	3
Somatic kineties, number	P	7.3	7.0	0.2	6.6	7.0	8.0	10
Kinetids in anterior part of cell, number	P	8.6	8.0	1.3	15.7	7.0	11.0	10
Kinetids in posterior part of cell, number	P	2.0	2.0	0.0	0.0	2.0	2.0	12
M1 to M3 distance	P	4.3	4.2	0.4	10.4	3.9	5.1	6
Distance between M3 and posterior end of the paroral	P	3.2	3.2	0.3	9.8	2.9	3.8	8
Apical plate, diameter	in vivo	3.4	3.4	0.4	10.6	3.1	3.6	2
Somatic cilia in anterior part of cell, length	in vivo	8.9	8.9	0.7	7.9	8.1	10.7	20
Caudal cilium, length	in vivo	20.0	20.8	1.5	7.6	17.5	21.2	5
Macronucleus, diameter	P	3.2	3.2	0.4	13.5	2.5	4.6	28
Macronuclear nodules, number	P	1.0	1.0	0.2	18.2	1.0	2.0	28
Micronucleus, diameter	P	1.4	1.4	0.2	14.7	1.1	1.9	21
Ectosymbionts, cell length	in vivo	4.1	3.9	0.8	18.8	3.0	5.4	15
	SEM	2.8	2.7	0.6	20.3	2.0	4.4	23

[a]All distances in µm. Measurements made using ocular micrometer.
CV, coefficient of variation (%); M, median; M1-3, adoral membranelle 1-3; Max, maximum value; Mean, arithmetic mean; Min. minimum value; *n*, number of cells studied; P, protargol; SD, standard deviation of the arithmetic mean; SEM, scanning electron microscopy.

Supplementary Table S3. Morphometric data for *Maricyclidium* lineage 2 (strain ALB1).

Characteristic[a]	Method	Mean	M	SD	CV	Min	Max	*n*
Cell length	in vivo	17.5	17	1.3	7.5	16	21	20
	P	19.0	19.3	1.3	6.8	16.5	21.4	42
Cell width	in vivo	8.8	9.0	1.0	11.4	7.0	11.0	20
	P	13.0	13.0	1.2	8.9	10.1	14.7	42
Cell length-width, ratio	in vivo	2.0	2.0	0.2	9.8	1.7	2.4	20
	P	1.5	1.5	0.1	4.9	1.3	1.7	42
Buccal region, length	P	10.3	10.2	0.6	6.2	9.1	11.6	32
Buccal region length/cell length %	P	60	50	0.1	10.6	40	70	32
Buccal region, width	P	2.3	2.2	0.3	12.1	1.9	3.0	23
Kinetids in anterior part of cell, number	P	9.1	9.0	0.7	8.0	8.0	11.0	30
Kinetids in posterior part of cell in SK2–SKn, number	P	2.0	2.0	0.0	0.0	2.0	2.0	18
Kinetids in posterior part of SK1, number	P	3.0	3.0	0.0	0.0	3.0	3.0	10
Somatic kineties, number	P	7.7	8.0	0.5	6.0	7.0	8.0	11
Distance between M3 and posterior end of paroral	P	3.8	3.8	0.3	7.3	3.3	4.2	7
Transverse distance between kineties	P	2.7	2.7	0.3	10.8	2.3	3.0	5
Apical plate, width	in vivo	3	3	0.0	0.0	3	3	20
Somatic cilia, length	in vivo	8.1		0.4	4.6	8	9	7
Caudal cilium, length	in vivo	16.3	16.0	1.3	7.7	15	18	4
Macronucleus, diameter	P	4.9	4.9	0.5	10.1	3.8	6.0	32
Macronuclei, number	P	1.0	1.0	0.0	0.0	1.0	1.0	32
Micronucleus, diameter	P	2.2	2.1	0.5	21.0	1.6	3.1	19
Micronuclei, number	P	1.0	1.0	0.0	0.0	1.0	1.0	21
Ectosymbionts, cell length	in vivo	4.4	4.4	0.5	12.2	3.6	5.4	15
	SEM	3.9	3.6	0.8	21.5	2.7	5.6	20

[a]All distances in μm. Measurements made using ocular micrometer.
CV, coefficient of variation (%); M, median; M3, adoral membranelle 3; Max, maximum value; Mean, arithmetic mean; Min, minimum value; *n*, number of cells studied; P, protargol; SD, standard deviation of the arithmetic mean; SEM, scanning electron microscopy; SK1, somatic kinety 1; SK2, somatic kinety 2; SKn, somatic kinety *n*.

Supplementary Table S4. Morphometric data for *Maricyclidium* lineage 3 (strain OCE20C).

Characteristic[a]	Method	Mean	M	SD	CV	Min	Max	*n*
Cell length	in vivo	18.0	18.0	1.0	5.8	16.7	20.8	17
	P	17.6	17.6	1.0	5.4	15.8	19.5	21
Cell width	in vivo	9.9	10.1	0.9	8.7	8.0	10.8	17
	P	11.1	10.8	1.2	10.4	8.9	13.7	21
Cell length-width, ratio	in vivo	1.8	1.8	0.2	9.1	1.6	2.2	17
	P	1.6	1.6	0.1	6.3	1.4	2.2	21
Buccal region, length	in vivo	10.0	10.1	0.4	4.1	9.3	10.6	16
	P	9.6	9.6	0.7	6.9	8.6	10.9	18
Buccal region length/cell length %	in vivo	60	60	0.0	6.5	50	60	16
	P	60	60	0.0	6.6	50	60	18
Buccal region, width	in vivo	2.9	3.0	0.2	8.5	2.4	3.3	12
	P	2.1	2.0	0.2	11.2	1.8	2.6	11
Somatic kineties, number	P	7.8	8.0	0.4	4.8	7.0	8.0	13
Kinetids in anterior part of somatic kinety, number	P	8.4	8.0	0.7	8.2	7.0	10.0	19
Kinetids in posterior part of somatic kinety, number	P	2.2	2.0	0.4	17.0	2.0	3.0	20
M1 to M3 distance	P	5.6	5.5	0.4	6.7	5.2	6.3	7
Distance between M3 and posterior end of the paroral	P	3.6	3.7	0.3	7.2	3.2	3.9	7
Transverse distance between mid-dorsal kineties	P	2.5	2.2	0.5	18.7	2.2	3.0	3
Apical plate, diameter	in vivo	4.5	4.7	0.4	8.1	3.9	4.9	12
Somatic cilia in posterior part of cell, length	in vivo	11.2	11.2	0.5	4.8	10.4	11.8	5
Caudal cilium, length	in vivo	20.1	20.3	2.0	10.0	17.1	23.5	11
Macronuclei, diameter	P	4.5	4.5	0.6	13.4	2.8	5.8	36
Macronuclear nodules, number	P	1.1	1.0	0.5	45.1	1.0	4.0	38
Micronucleus, diameter	P	1.9	1.9	0.3	16.8	1.1	2.5	28
Ectosymbionts, cell length	in vivo	4.7	4.5	0.9	19.6	3.7	7.2	21

[a]All distances in µm. Measurements made using ocular micrometer.
CV, coefficient of variation (%); M, median; M1-M3, adoral membranelle 1-3; Max, maximum value; Mean, arithmetic mean; Min. minimum value; *n*, number of cells studied; P, protargol; SD, standard deviation of the arithmetic mean.

Supplementary Table S5. Morphometric data for *Maricyclidium* lineage 4 (strain CRVAR2B).

Characteristic[a]	Method	Mean	M	SD	CV	Min	Max	*n*
Cell length	in vivo	20.7	20.6	1.6	7.6	17.2	22.8	26
	P	17.3	16.8	1.5	8.9	14.5	19.7	18
Cell width	in vivo	10.5	10.8	1.2	11.5	8.0	12.5	26
	P	7.0	6.8	0.8	11.6	5.6	9.2	18
Cell length-width, ratio	in vivo	2.0	2.0	0.2	9.5	1.7	2.6	26
	P	2.5	2.5	0.2	8.0	2.1	3.0	18
Buccal region, length	in vivo	10.8	10.8	0.6	5.5	9.9	11.9	19
	P	9.1	9.2	0.7	7.2	8.3	10.2	11
Buccal region length/cell length %	in vivo	50	50	0.0	7.7	50	60	19
	P	50	50	0.0	9.0	50	60	11
Buccal region, width	in vivo	2.7	2.8	0.3	9.3	2.3	3.1	6
Somatic kineties, number	P	7.6	8.0	0.5	6.8	7.0	8.0	14
Kinetids in anterior part of somatic kinety, number	P	8.1	8.0	0.7	8.4	7.0	9.0	16
Kinetids in posterior part of somatic kinety, number	P	2.4	2.0	0.5	21.1	2.0	3.0	15
Transverse distance between kineties	P	2.5	2.5	0.2	6.0	2.4	2.7	4
Apical plate, width	in vivo	4.5	4.4	0.5	11.6	3.6	5.3	10
Somatic cilia, length	in vivo	9.4	9.3	0.7	7.0	8.5	10.6	10
Caudal cilium, length	in vivo	22.1	21.8	2.3	10.5	19.5	25.7	5
Macronucleus, diameter	P	4.3	4.2	0.4	9.8	3.7	5.2	18
Macronuclear nodules, number	P	1.0	1.0	0.0	0.0	1.0	1.0	18
Micronucleus, diameter	P	1.3	1.3	0.2	17.0	0.9	1.9	18
Micronuclei, number	P	1.2	1.0	0.4	32.9	1.0	2.0	18
Ectosymbionts, cell length	SEM	6.4	6.3	0.8	12.7	5.3	8.2	11

[a]All distances in µm. Measurements made using ocular micrometer.
CV, coefficient of variation (%); M, median; Max, maximum value; Mean, arithmetic mean; Min, minimum value; *n*, number of cells studied; P, protargol; SD, standard deviation of the arithmetic mean.

Supplementary Table S6. Morphometric comparison of *Maricyclidium* strains (in vivo).

Characteristic[a]	Mean	M	SD	CV	Min	Max	*n*
Cell length							
Maricyclidium commune strain SIP1E	19.7	19.5	1.10	5.5	18.0	21.0	22
Maricyclidium lineage 2 strain ALB1	17.5	17.0	1.3	7.5	16.0	21.0	20
Maricyclidium lineage 3 strain OCE20C	18.0	18.0	1.0	5.8	16.7	20.8	17
Maricyclidium lineage 4 strain	20.7	20.6	1.6	7.6	17.2	22.8	26
Maricyclidium lineage 1 strain	18.3	17.7	1.3	7.0	16.7	20.9	20
Cell width							
Maricyclidium commune strain SIP1E	10.0	10.0	0.7	6.6	9.0	11.0	22
Maricyclidium lineage 2 strain ALB1	8.8	9.0	1.0	11.4	7.0	11.0	20
Maricyclidium lineage 3 strain OCE20C	9.9	10.1	0.9	8.7	8.0	10.8	17
Maricyclidium lineage 4 strain	10.5	10.8	1.2	11.5	8.0	12.5	26
Maricyclidium lineage 1 strain	10.6	10.6	0.7	6.8	8.6	11.7	20
Cell length-width, ratio							
Maricyclidium commune strain SIP1E	2.0	2.0	0.1	4.7	1.8	2.1	22
Maricyclidium lineage 2 strain ALB1	2.0	2.0	0.2	9.8	1.7	2.4	20
Maricyclidium lineage 3 strain OCE20C	1.8	1.8	0.2	9.1	1.6	2.2	17
Maricyclidium lineage 4 strain	2.0	2.0	0.2	9.5	1.7	2.6	26
Maricyclidium lineage 1 strain	1.7	1.8	0.1	6.5	1.5	1.9	20
Paroral length:cell length, %							
Maricyclidium commune strain SIP1E	50.0	50.0	0.0	4.9	50.0	60.0	18
Maricyclidium lineage 2 strain ALB1	–	–	–	–	–	–	–
Maricyclidium lineage 3 strain OCE20C	60.0	60.0	0.0	6.5	50.0	60.0	16
Maricyclidium lineage 4 strain	50.0	50.0	0.0	7.7	50.0	60.0	19
Maricyclidium lineage 1 strain	–	–	–	–	–	–	–
Apical plate, width							
Maricyclidium commune strain SIP1E	3.8	4.0	0.4	10.6	3.0	4.0	16
Maricyclidium lineage 2 strain ALB1	3.0	3.0	0.0	0.0	3.0	3.0	20
Maricyclidium lineage 3 strain OCE20C	4.5	4.7	0.4	8.1	3.9	4.9	12
Maricyclidium lineage 4 strain	4.5	4.4	0.5	11.6	3.6	5.3	10
Maricyclidium lineage 1 strain	3.9	4.0	0.63	16.4	3.0	5.0	14
Somatic cilia, length							
Maricyclidium commune strain SIP1E	9.2	9.1	0.9	9.7	7.9	10.9	15
Maricyclidium lineage 2 strain ALB1	8.1	8.0	0.4	4.6	8.0	9.0	7
Maricyclidium lineage 3 strain OCE20C	11.2	11.2	0.5	4.8	10.4	11.8	5
Maricyclidium lineage 4 strain	9.4	9.3	0.7	7.0	8.5	10.6	10
Maricyclidium lineage 1 strain	8.9	8.9	0.7	7.9	8.1	10.7	20
Caudal cilium, length							
Maricyclidium commune strain SIP1E	17.6	17.0	1.4	7.8	16.0	20.0	12
Maricyclidium lineage 2 strain ALB1	16.3	16.0	1.3	7.7	15.0	18.0	4
Maricyclidium lineage 3 strain OCE20C	20.1	20.3	2.0	10.0	17.1	23.5	11
Maricyclidium lineage 4 strain	22.1	21.8	2.3	10.5	19.5	25.7	5
Maricyclidium lineage 1 strain	20.0	20.8	1.5	7.6	17.5	21.2	5

[a]All distances in µm. Measurements made using ocular micrometer.
CV, coefficient of variation (%); M, median; Max, maximum value; Mean, arithmetic mean; Min. minimum value; *n*, number of cells studied; SD, standard deviation of the arithmetic mean.

Supplementary Table S8. Results of approximately unbiased test of constraint trees representing competing hypotheses regarding taxa discussed herein and whose monophyly was tested. Hypotheses in which p<0.05 are rejected as monophyletic and highlighted in gray.

**Hypothesis tested**	**-logL**	**p-AU**
Unconstrained tree	39377.69153	1.000
Neocyclidium + *Metacyclidium* monophyletic	39714.78018	3.39e-06
Neocyclidium + *Anaerocyclidium* monophyletic	39684.97169	4.89e-43
Metacyclidium + *Anaerocyclidium* monophyletic	39722.05527	1.18e-117
