# Supplementary material for: Capturing the extensive diversity of marine anaerobic scuticociliates (Oligohymenophorea, Ciliophora) through cultivation
Source: Mar Life Sci Technol. 2026 Mar 30;8(2):256–75. doi: 10.1007/s42995-025-00350-5 (PMC13198587; doi:10.1007/s42995-025-00350-5)
Supplement: Supplementary file 3 — Supplementary file3 (PDF 1900 KB) [file 42995_2025_350_MOESM3_ESM.pdf]

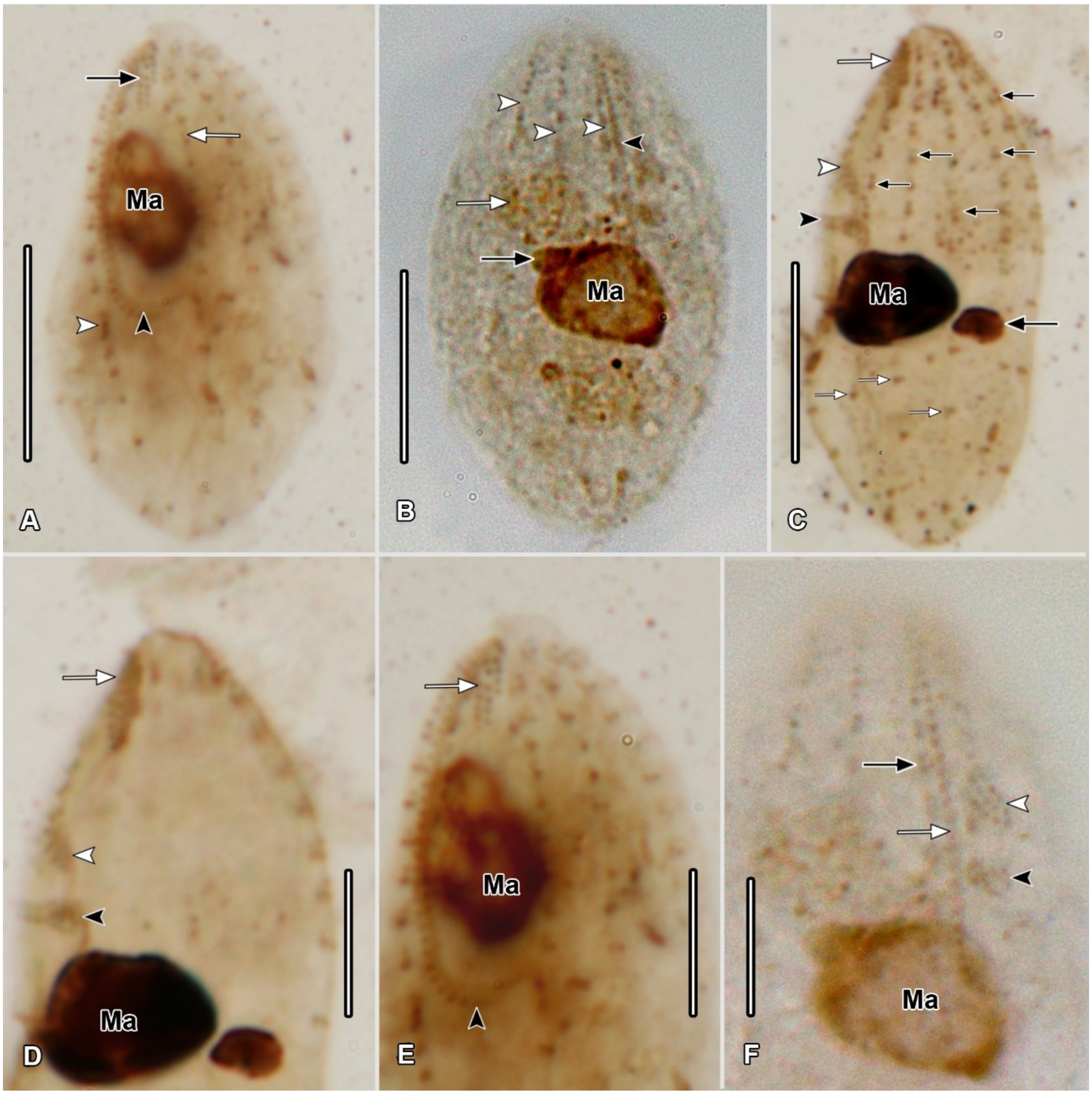

Supplementary Fig. S1 *Neocyclidium profundum* n. gen., n. sp. after protargol impregnation (A–G). **A** Ventral view showing adoral membranelle 1 (M1, black arrow), somatic kinety 1 (SK1, white arrowhead), SKn (white arrow), and posterior paroral margin (black arrowhead). **B** Right ventrolateral view showing food vacuole (white arrow), anterior somatic kineties (white arrowheads), paroral (black arrowhead), and micronucleus (black arrow). **C, D** Left lateral view (C) and close-up (D) showing M1 (white arrow), M2 (white arrowhead), M3 (black arrowhead), anterior dikinetids (small black arrows), posterior monokinetids (small white arrows), and micronucleus (black arrow). **E** Detail of M1 (white arrow), M2 (white arrowhead), M3 (black arrowhead). **F** Detail of longitudinal files of M1 (white arrow) and posterior margin of stichodyad paroral (black arrowhead). **G** Detail showing SK1 (black arrow) close to paroral (white arrow), triangular M2 basal bodies (white arrowhead), and rectangular M3 (black arrowhead). Ma, macronucleus. Scale bars: 10 µm (A–C), 5 µm (D–G).

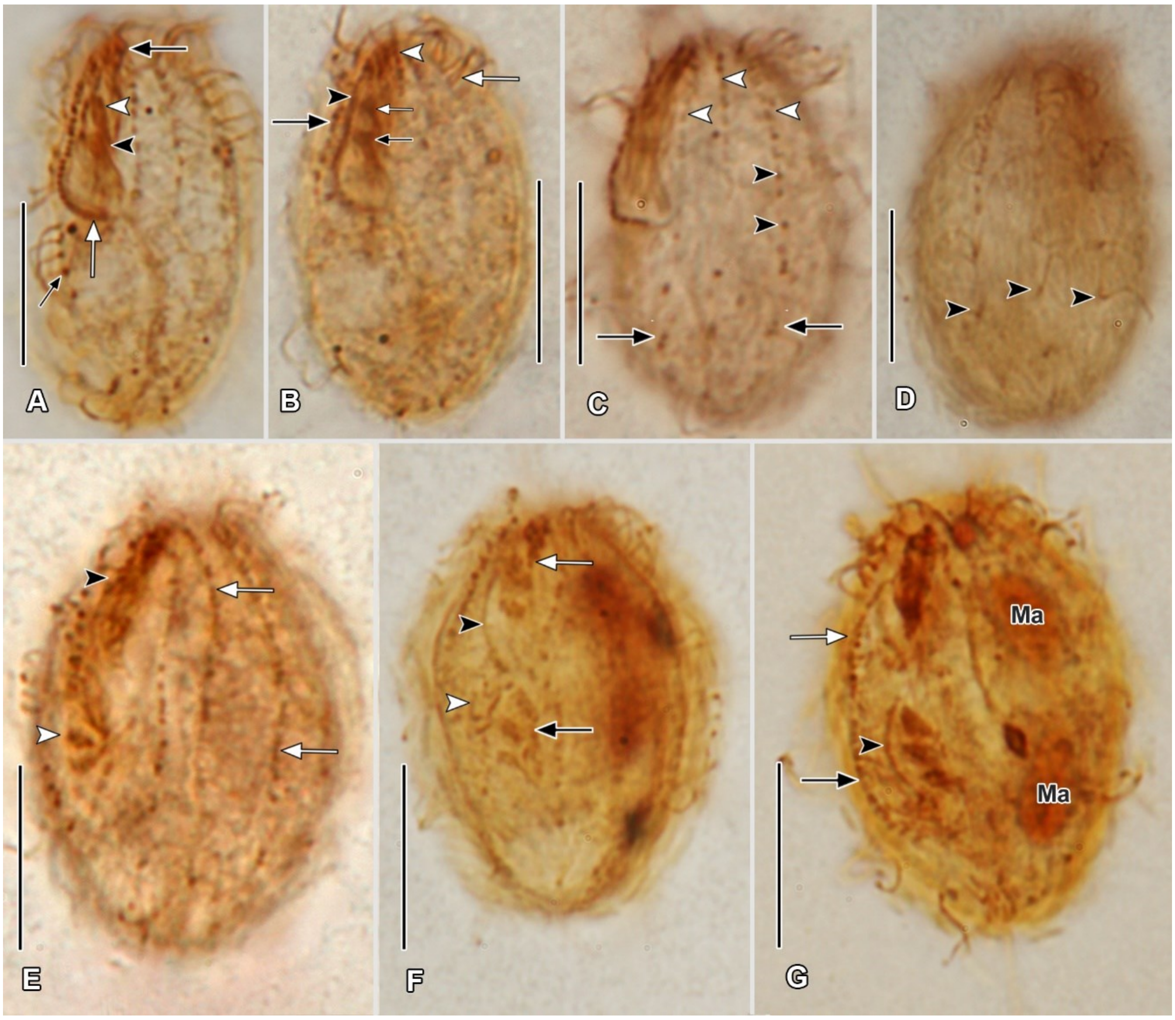

Supplementary Fig. S2 *Metacyclidium pallium* n. gen., n. sp. after protargol impregnation (**A–G**). **A** Left ventrolateral view showing posterior paroral margin (white arrow), closely spaced monokinetids in mid SK1 (small black arrow), and adoral membranelles M1 (black arrow), M2 (white arrowhead), M3 (black arrowhead). **B** Optical section showing ciliated dikinetids in anterior SK (white arrow), SK1 basal bodies (black arrow), paroral (black arrowhead), and M1 (white arrowhead), M2 (small white arrow), M3 (small black arrow). **C** Left ventrolateral view showing closely spaced anterior dikinetids (white arrowheads), widely spaced posterior dikinetids (black arrows), and mid monokinetids (black arrowheads). **D** Right lateral view showing ciliation of posterior dikinetids (black arrowheads). **E** Divider showing intrakinetal basal body proliferation (white arrows) and developing oral structures of proter (black arrowhead) and opisthe (white arrowhead). **F** Divider with developing adoral membranelles of proter (white arrow) and opisthe (black arrow); proter paroral (black arrowhead) reforms before opisthe paroral (white arrowhead). **G** Divider showing developing SK1 in proter (white arrow) and opisthe (black arrow) and opisthe paroral (black arrowhead). Ma, macronucleus. Scale bars: 10 µm.

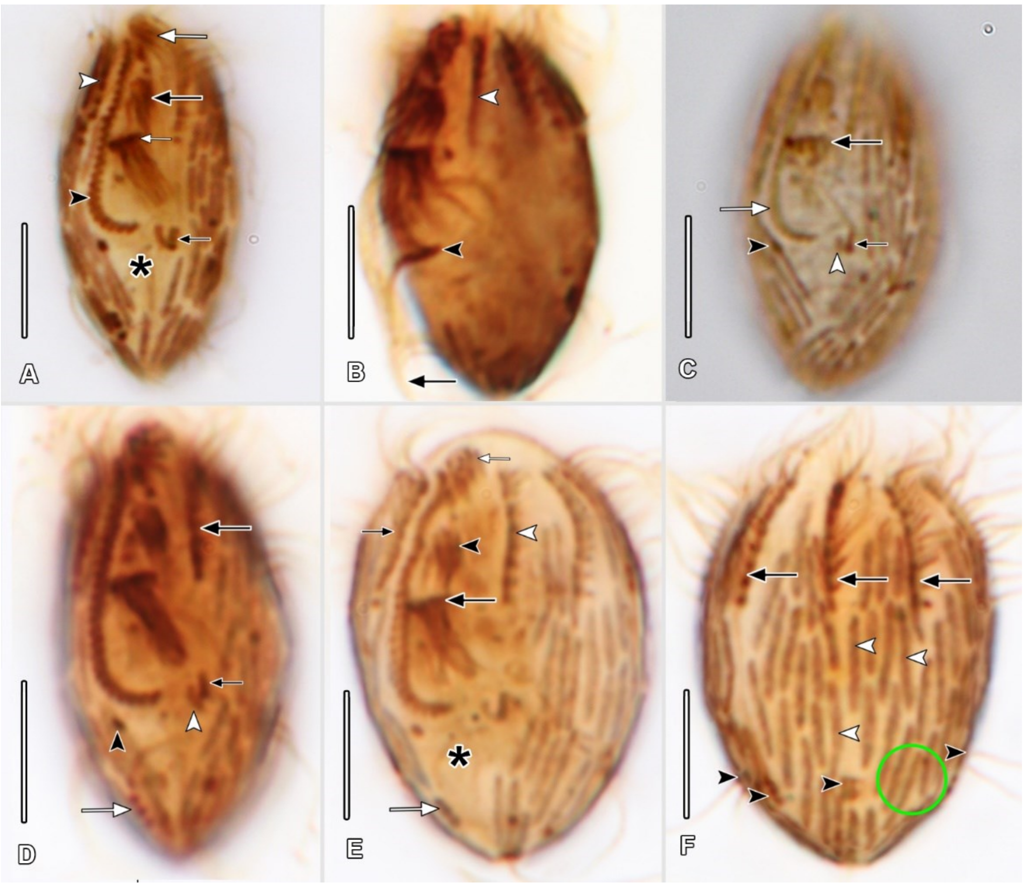

Supplementary Fig. S3 *Maricyclidium commune* n. gen., n. sp. after protargol impregnation (**A–F**). **A** Ventral view showing M1 (white arrow), M2 (black arrow), M3 (small white arrow), anterior SK1 (white arrowhead), stichodyad paroral (black arrowhead), postoral bare area (asterisk), and posterior SKn (small black arrow). **B** Optical section showing posterior paroral margin (black arrowhead), paroral cilia (black arrow), and anterior SKn (white arrowhead). **C** Ventral view showing paroral (white arrow), right (black arrowhead) and left (white arrowhead) scutica, posterior SKn (small black arrow), and M3 (black arrow). **D** Ventral view showing three basal bodies of posterior SK1 (white arrow), anterior (black arrow) and posterior (small black arrow) SKn, and right (black arrowhead) and left (white arrowhead) scutica basal bodies. **E** Ventral view showing M1 (small white arrow), M2 (black arrowhead), M3 (black arrow), anterior SK1 (small black arrow) and SKn (white arrowhead), posterior SK1 (white arrow), and postoral bare area (asterisk). **F** Right dorsolateral view showing anterior (black arrows) and posterior (black arrowheads) SK fragments and cortical ectosymbionts (white arrowheads). SK3 lacks a posterior fragment (green circle). Scale bars: 5 µm.

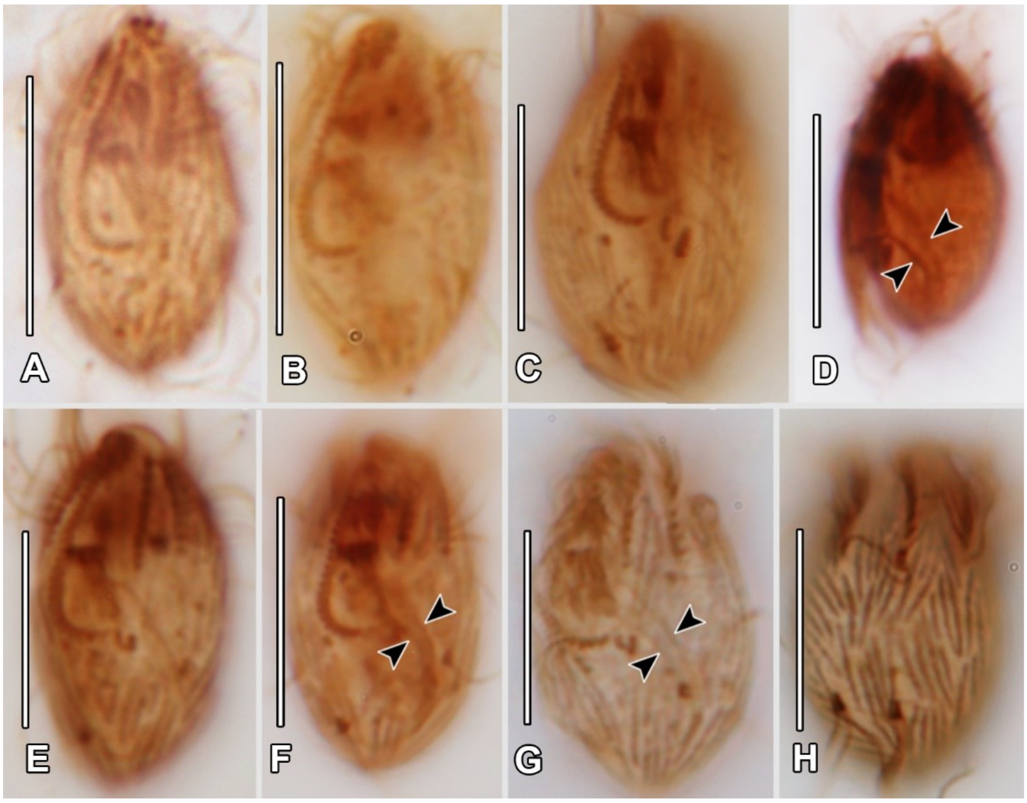

Supplementary Fig. S4 Undescribed *Maricyclidium* species: *Maricyclidium* lineage 1 (strain LJUBACKI, **A**, **B**), *M.* lineage 3 (strain OCE20C, **C**, **D**), *M.* lineage 2 (strain ALB1, **E**, **F**), and *M.* lineage 4 (strain CRVAR2B, **G**, **H**) after protargol impregnation. **A, B** Ventral views showing buccal and somatic infraciliature. **C, D** Ventral view of buccal and somatic infraciliature (C) and optical section (D) showing oral basket (black arrowheads). **E, F** Ventral view of infraciliature (E) and optical section showing oral basket (black arrowheads) (F). **G** Ventral view of buccal and somatic infraciliature showing the oral basket (black arrowheads). **H** Dorsal view of infraciliature and ectosymbionts. Scale bars: 10 µm.

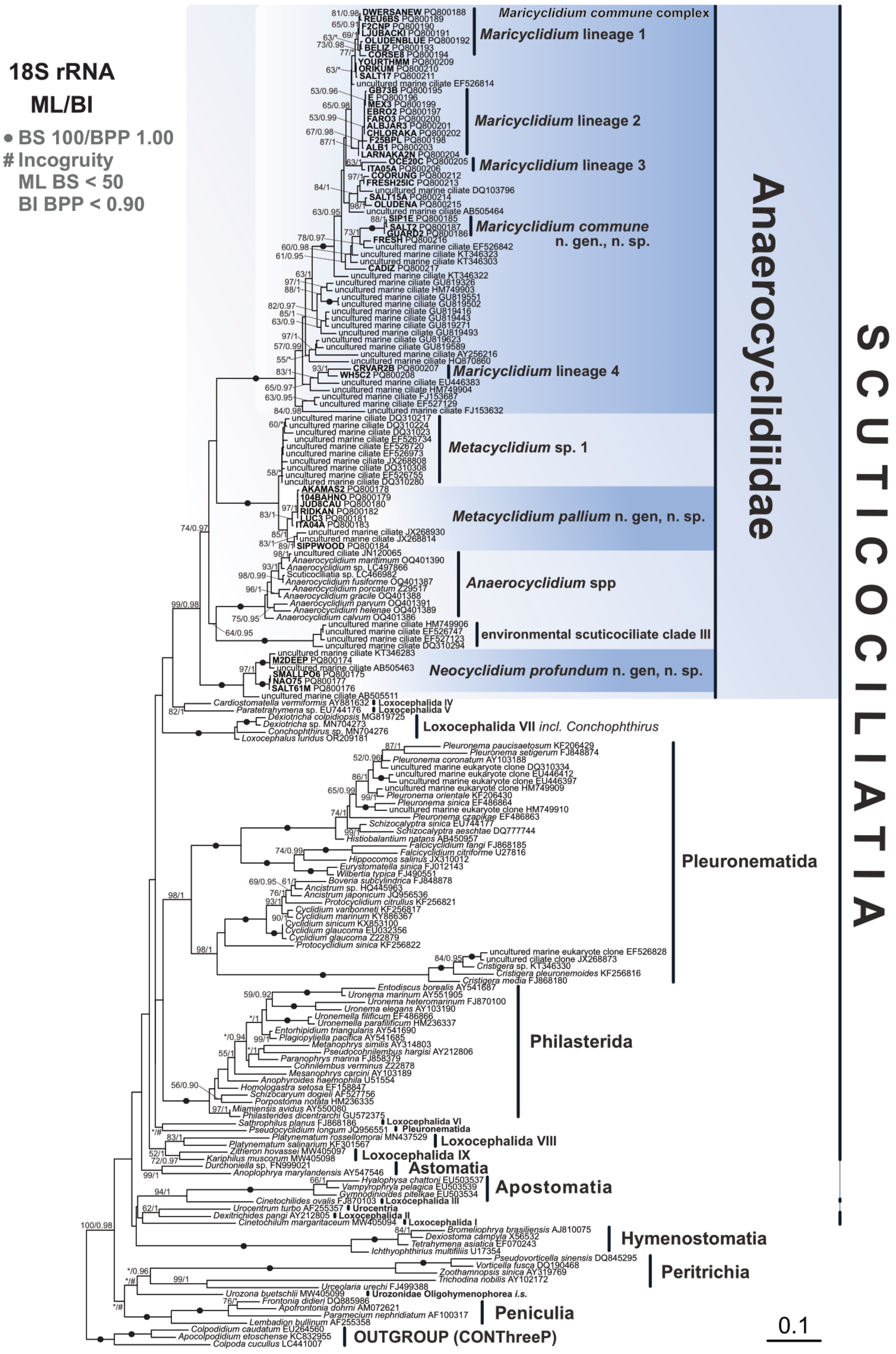

Supplementary Fig. S5 Full version of the phylogenetic tree depicted in Fig. 6.
